# Optimization of segmented thermoelectric generator using Taguchi and ANOVA techniques

**DOI:** 10.1038/s41598-017-16372-8

**Published:** 2017-12-01

**Authors:** Ravi Anant Kishore, Mohan Sanghadasa, Shashank Priya

**Affiliations:** 1 Center for Energy Harvesting Materials and Systems (CEHMS), Virginia Tech, Blacksburg, VA 24061 USA; 2 Aviation and Missile Research, Development, and Engineering Center, US Army RDECOM, Redstone Arsenal, AL 35898 USA

## Abstract

Recent studies have demonstrated that segmented thermoelectric generators (TEGs) can operate over large thermal gradient and thus provide better performance (reported efficiency up to 11%) as compared to traditional TEGs, comprising of single thermoelectric (TE) material. However, segmented TEGs are still in early stages of development due to the inherent complexity in their design optimization and manufacturability. In this study, we demonstrate physics based numerical techniques along with Analysis of variance (ANOVA) and Taguchi optimization method for optimizing the performance of segmented TEGs. We have considered comprehensive set of design parameters, such as geometrical dimensions of p-n legs, height of segmentation, hot-side temperature, and load resistance, in order to optimize output power and efficiency of segmented TEGs. Using the state-of-the-art TE material properties and appropriate statistical tools, we provide near-optimum TEG configuration with only 25 experiments as compared to 3125 experiments needed by the conventional optimization methods. The effect of environmental factors on the optimization of segmented TEGs is also studied. Taguchi results are validated against the results obtained using traditional full factorial optimization technique and a TEG configuration for simultaneous optimization of power and efficiency is obtained.

## Introduction

Thermoelectric generators (TEGs) are currently the most pursued thermal energy harvesting technology. Solid-state structure with no moving parts or harmful chemical discharge makes TEGs reliable, maintenance-free, and noiseless. TEGs utilize Seebeck effect in order to convert heat directly into electricity^1^. Despite these salient features, TEGs have been used only in limited practical applications^2–4^. One of the key reasons behind this is the low figure of merit (ZT) of thermoelectric (TE) materials. The figure of merit (ZT) is defined as $ZT=\frac{{S}^{2}T}{\rho \kappa }$, where *S* is the Seebeck coefficient, ρ is the electrical resistivity, κ is the total thermal conductivity, and T is the absolute temperature^5^. There have been extensive studies conducted in the last few decades in order to enhance the ZT of TE materials by increasing the power factor $(\frac{{S}^{2}}{\rho })$ and by reducing the thermal conductivity (κ). Some techniques, such as nanostructuring, doping with Cu or Ag atoms, and adjusting atomic ratios, have been found to improve the ZT of TE materials^6–14^. A recent study by Hu *et al*. demonstrated that doping p-type nanostructured PbTe based TE material with 4% Na reduces lattice thermal conductivity through nanostructuring and results in ZT of 1.8 at 810 K^15^. Likewise, doping n-type nanostructured PbTe with 0.2% PbI_2_ provided ZT of 1.4 at 750 K^15^. The TEG module fabricated with these materials were found to have maximum thermal-to-electrical energy conversion efficiency of 8.8% at a temperature difference of 570 K^15^.

Some of the state-of-the-art TE materials reported in literature are quantum-dot superlattice with ZT of 3.5 at 575 K by Harman *et al*.^16^, thin film superlattice structure with ZT of 2.4 at 300 K and ZT of 2.9 at 400 K by Venkatasubramanian *et al*.^17^, and lead antimony silver telluride (AgPb_m_SbTe_2+m_) with ZT of 2.2 at 800 K by Hsu *et al*.^18^. Nonetheless, these excellent laboratory results have not been transitioned into practical applications^19^. It is also important to note that ZT is a highly temperature dependent parameter. This implies that even though a relatively higher value of ZT is reported in a localized temperature range, the average-ZT in a wide operating range for the commercially available TE modules is still close to unity^19,20^. The most common TE materials currently used are BiTe alloys (temperature: 100–200 °C)^21–26^, PbTe alloys (temperature: 350–650 °C)^5,18,27–35^, skutterudites (temperature: 300–600 °C)^36–43^, half-heusler alloys (temperature: 500–800 °C)^44,45^, and Si-Ge alloys (temperature: 900–950 °C)^46,47^. Few researchers have suggested combining different TE materials to build segmented TEGs^15,48,49^. Segmented TEGs consist of two or more layers of TE materials arranged in series. Segmentation, therefore, allows TEGs to operate in a larger thermal gradient thereby providing higher output power and efficiency compared to the non-segmented TEGs under the same thermal gradient. The study by Hu *et al*. reported that a segmented TEG module built using nanostructured PbTe- and BiTe-based materials had efficiency of 11% at temperature difference of 590 K, as compared to efficiency of 8.8% from a non-segmented TEG module made using just nanostructured PbTe material^15^.

Despite the promising results demonstrated by segmented TEGs, they have not been extensively investigated due to inherent complexity in their design optimization and manufacturability. Segmentation introduces additional thermal and electrical interfaces between different TE layers, which increases contact resistances. The electrical contact resistance generates extra Joule heat and the thermal resistance leads to abrupt temperature drop at the interface. Both these effects are undesired as they adversely affect the performance of TEGs. The performance of TEGs not only depends on the ZT of TE materials but also on the configuration of the TEG modules. Geometric parameters such as length, width, and height of p-n legs, gap distance between legs, operating conditions such as hot-side and cold-side temperatures, and energy losses due to convection and radiation, collectively affect performance of TEGs. Optimizing all the performance parameters using conventional modeling techniques, where only one factor is changed at a time, requires several experiments. This makes the optimization process cumbersome, time-consuming, and expensive. The complexity further increases by adding additional layers of TE materials due to segmentation. In this study, we utilize numerical techniques along with Analysis of variance (ANOVA) and Taguchi optimization method to design segmented TEGs. We have used material properties of nanostructured PbTe and BiTe reported in reference^15^. ANOVA is a statistical method that allows us to estimate the relative significance of different process parameters that affect the performance of system. In order to reduce the design optimization cost, we implement Taguchi method of optimization. Taguchi method is an established statistical optimization technique that uses certain orthogonal arrays to predict the optimal performance with far less number of experimental runs than the conventional optimization techniques, where only one factor is normally changed at a given instance. Taguchi method allows us to vary multiple factors at the same time in a controlled manner, thereby reducing the total number of experimental runs required^50^. Although this method was originally developed for manufacturing industries to optimize product quality and production cost, it is now widely used in the diverse field of research and engineering^51–60^. However, this method has not been much employed for designing high performance thermoelectric modules. Chen *et al*. used Taguchi method to optimize the dimensions, length, width, and height, of the heat sink for TEG with respect to given hot-side temperature and resistive load^61^. Kishore *et al*. performed Taguchi optimization on thermoelectric cooler (TEC) to optimize the p-n legs dimensions and to study the effect of environmental factors on TEC optimization^62^.

We have achieved optimization of segmented TEGs in the following manner. First, we validate the numerical model using published experimental data and study the effect of contact resistances on the output power and efficiency. Next, geometric parameters of the segmented TEG, namely cross-sectional area, segmented height and total height of the p-n legs, along with the resistive load and hot-side temperature are optimized using Taguchi method. We also validate the Taguchi results against the data obtained from conventional full factorial optimization method. We then study the effect of noise factors, namely ambient temperature and cooling coefficient, on the optimization of segmented TEG. Lastly, we propose setting for simultaneous optimization of power and efficiency of the segmented TEGs. The study reveals that Taguchi method can effectively optimize segmented TEG and predict the optimal geometric parameters along with operating conditions with far less number of experiments than the conventional techniques.

## Results and Discussion

**Figure 1:**
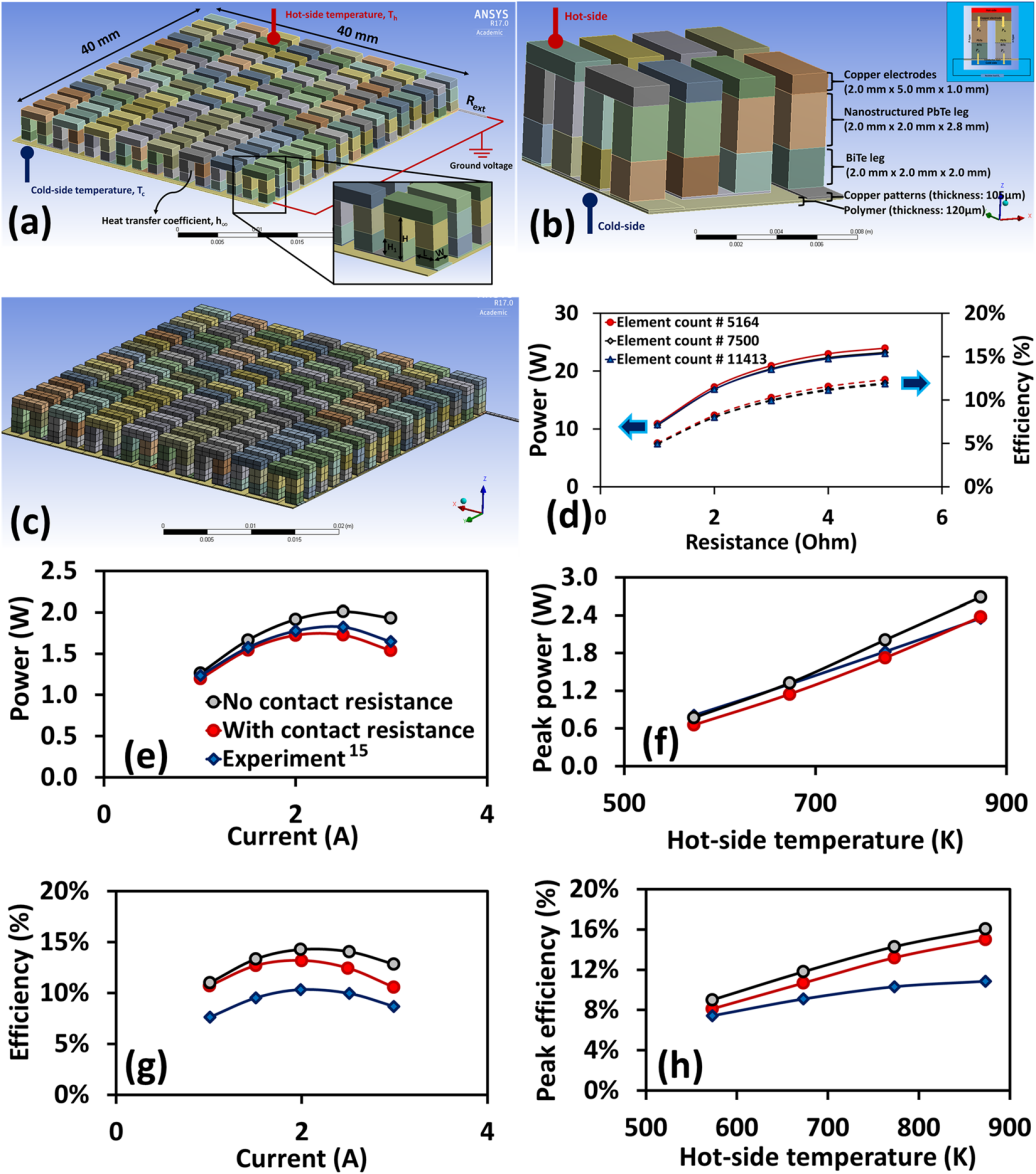
The computational model of segmented TEG. (**a**) CAD model of the segmented TEG with key geometric parameters and boundary conditions used for numerical simulations. (**b**) CAD model of the segmented TEG used for numerical validation. (**c**) A medium-size grid structure used for the simulations, element type: SOLID226 (3D 20-node hexahedron/brick). (**d**) Mesh independency test, comparing output power and efficiency at three different element counts. (**e**) Power output vs. electrical current at different contact resistance conditions (hot-side and cold-side temperatures are fixed at T_h_ = 773 K and T_c_ = 283 K). (**f**) Peak power vs. hot-side temperature at different contact resistance conditions. (**g**) Efficiency vs. electrical current at different contact resistance conditions (hot-side and cold-side temperatures are fixed at T_h_ = 773 K and T_c_ = 283 K). (**f**) Peak efficiency vs. hot-side temperature at different contact resistance conditions.

Figure 1(a) shows the three-dimensional CAD model of the segmented TEG module developed in ANSYS designModeler. The key geometric parameters are also indicated in the inset. The overall cross-sectional area of the TEG module is fixed at 40 × 40 mm^2^. The segmented p-n legs consist of p-type and n-type PbTe and p-type and n-type BiTe legs connected electrically in series and thermally in parallel. The height of p and n sections of each material is taken to be equal. Likewise, the length, L, and width, W, of the p-n legs are considered equal and varied together in the range of 1.5 × 1.5 mm^2^–2.5 × 2.5 mm^2^. The height of BiTe portion, H_1_, and total height, H, of the p-n legs are varied in the range of 1.25 mm–2.25 mm and 3.5 mm–5.0 mm, respectively. The gap distance between p-n legs is fixed at 1.0 mm. The top copper electrode on hot-side has thickness of 1.0 mm, whereas copper patterns on the bottom towards the cold-side has thickness of 0.105 mm. A heat conducting polymer film of thickness 0.120 mm is provided at the bottom to electrically insulate the TEG module. The material properties of all the materials including heat conducting polymer film are taken from reference^15^. In order to validate the numerical model, we also built a segmented TEG of the same dimensions as reported in ref.^15^ and compared the numerical results with the experimental results. As shown in Fig. 1(b), this module consists of eight pairs of p-n legs having cross-sectional area of 2.0 mm × 2.0 mm and the total height of 4.8 mm. The BiTe legs have dimension of 2.0 mm × 2.0 mm × 2.0 mm and nanostructured PbTe legs have dimension of 2.0 mm × 2.0 mm × 2.8 mm.

We used SOLID226 (a 3D 20-node hexahedron/brick) elements to discretize the FEA model. Mesh independency test was performed to ensure that the numerical results are independent of the grid size. Figure 1(c) shows a medium-size grid structure, having element count of 7500 and node count of 73459. Figure 1(d) compares the power and efficiency values obtained using three different grid densities. It was noted that the maximum difference in the results from coarse (element count # 5164) to medium (element count # 7500) size mesh was around 3.5%; whereas difference in the results from medium (element count # 7500) to fine (element count # 11413) size mesh was less than 1.0%. Therefore, for all the simulations in this study, we have used medium size meshing strategy.

Figure 1(e–h) compare the experimental results with the numerical results. It can be observed that contact resistances play very important role in determining the performance of TEG. In case of segmented TEG, the contact resistances occur at the interface between different TE layers, between p-n legs and copper electrodes, and between copper electrodes and electrical insulator. The electrical contact resistance, ${\rho }_{ec}$, typically lies in the range of 1.0 × 10^−9^ to 1.0 × 10^−7^ Ω-m^2^, whereas, the thermal contact resistance, ${\rho }_{tc}$, has been reported to have typical values in the range of 1.0 × 10^−6^ to 1.0 × 10^−4^ m^2^-K-W^−1^ ^63–66^. TEG performance is highest when contact resistances are minimal. Output power and efficiency decreases with increase in either electrical contact resistance or thermal contact resistance or both. Figure 1(e) and (f) shows output power vs. electric current at given hot-side temperature, T_h_ = 773 K and peak power vs. hot-side temperature, respectively. Cold-side temperature is fixed at T_c_ = 283 K. Similarly, Fig. 1(g) and (h) show efficiency vs. electric current at hot-side temperature, T_h_ = 773 K and peak efficiency vs. hot-side temperature, respectively. By gradually varying the electrical and thermal contact resistances, it was found that at ${\rho }_{ec}$ = 5.0 × 10^−8^ Ω-m^2^ and ${\rho }_{tc}$ = 5.0 × 10^−4^ m^2^-K-W^−1^, and numerical and experimental results for power were within 14% range at all temperatures and electric currents. However, difference in numerical and experimental results for efficiency was large at high temperature, possibly due to the effect of diffusion barriers used in the experimental modules that affects the heat flow through the module^15^.

Table 1 shows the process factors and their levels considered in this study. The process factors that affect the performance of a system are of two types: (i) control factors and (ii) noise factors. Control factors can be controlled and adjusted; whereas, the noise factors are uncontrollable factors that occur due to environmental or external effects. The key control factors that affect the power output and efficiency of a segmented TEG are geometric parameters of the p-n legs, operating temperature, and external resistive load. On the other hand, some of the noise factors can be ambient temperature, atmospheric pressure, humidity and wind speed. Cold-side temperature is fixed at 283 K for all the simulations in this study.

**Table 1:** Process factors and their levels considered in this study for the optimization of segmented TEG.

Control factors		Levels				
		(1)	(2)	(3)	(4)	(5)
(A)	Hot-side temperature, T_h_ (K)	473	573	673	773	873
(B)	BiTe p-n leg height, H_1_ (mm)	1.25	1.5	1.75	2.0	2.25
(C)	Total p-n leg height, H (mm)	3.0	3.5	4.0	4.5	5.0
(D)	Cross-sectional area, A (mm^2^)	1.5 × 1.5	1.75 × 1.75	2.0 × 2.0	2.25 × 2.25	2.5 × 2.5
(E)	Resistive load, R (Ω)	1.0	2.0	3.0	4.0	5.0
**Noise factors**						
(F)	Ambient conditions, T_∞_ (K)	285	295	305	—	—
(G)	Cooling coefficient, h_∞_ (W/m^2^-K)	0	5	10	15	20

In Taguchi optimization method, control factors are varied according to certain standard orthogonal arrays (OAs). In this study, we have considered five factors at each of the five levels, therefore L_25_ orthogonal array is chosen whose structure is shown in Table S1 in supplementary document. It can be noted that with five factors at five levels, the traditional optimization method requires 5^5^ = 3125 experiments; whereas, Taguchi method, per L_25_ orthogonal array, needs only 25 experiments to predict the optimal output.

Table 2 shows output power and efficiency of the segmented TEG obtained using numerical simulations performed per Taguchi’s L_25_ orthogonal array. “Larger is better” Signal-to-noise (S/N) ratios for power, P, and efficiency, η, are also shown which are calculated using:

1 $${\rm{Larger}}\,{\rm{is}}\,{\rm{better}}\,S/N(dB)=-10\,\mathrm{log}[\frac{1}{r}\sum _{i=1}^{r}\frac{1}{{y}_{i}^{2}}]$$

where *r* is the number of data points and *y*_*i*_ is the value of the *i* ^*th*^ data point. Signal-to-noise (S/N) ratio is a very important concept in Taguchi optimization method. Greater the value of S/N ratio, larger is the effect of control factors over the noise factors on the output response. Depending on the goal of the optimization, Larger is better S/N ratios, Smaller is better S/N ratios, and Nominal is best S/N ratios, can be used. More details are provided in Supplementary information.

**Table 2:** Power (P), efficiency (η), and “larger is better” signal-to-noise ratios (S/N)_P_ and (S/N)η.

Trial 1	Control factors					Power, P (W)	(S/N)_P_ (dB)	Efficiency, η (%)	(S/N)_η_ (dB)
	(A)	(B)	(C)	(D)	(E)				
1	1	1	1	1	1	2.2436	7.0190	2.65%	−31.5209
2	1	2	2	2	2	3.3567	10.5183	4.71%	−26.5424
3	1	3	3	3	3	2.8050	8.9587	5.05%	−25.9414
4	1	4	4	4	4	2.8042	8.9564	4.66%	−26.6397
5	1	5	5	5	5	1.5026	3.5371	3.42%	−29.3105
6	2	1	2	3	4	6.129	15.7480	6.77%	−23.3911
7	2	2	3	4	5	6.0224	15.5954	6.21%	−24.1435
8	2	3	4	5	1	6.5025	16.2616	7.69%	−22.2816
9	2	4	5	1	2	3.7322	11.4394	4.73%	−26.4938
10	2	5	1	2	3	8.9964	19.0814	7.60%	−22.3791
11	3	1	3	5	2	10.911	20.7577	9.52%	−20.4303
12	3	2	4	1	3	8.4952	18.5834	7.54%	−22.4572
13	3	3	5	2	4	9.9901	19.9914	10.08%	−19.9298
14	3	4	1	3	5	11.7608	21.4087	8.70%	−21.2102
15	3	5	2	4	1	14.3355	23.1282	8.20%	−21.7236
16	4	1	4	2	5	16.5788	24.3910	12.51%	−18.0533
17	4	2	5	3	1	10.7442	20.6235	8.34%	−21.5717
18	4	3	1	4	2	27.1888	28.6878	11.68%	−18.6483
19	4	4	2	5	3	16.4796	24.3389	10.71%	−19.4062
20	4	5	3	1	4	14.7369	23.3681	9.45%	−20.4950
21	5	1	5	4	3	25.5562	28.1499	14.78%	−16.6059
22	5	2	1	5	4	21.9325	26.8217	10.90%	−19.2478
23	5	3	2	1	5	25.0300	27.9692	11.98%	−18.4295
24	5	4	3	2	1	12.6086	22.0133	6.08%	−24.3182
25	5	5	4	3	2	20.0729	26.0522	12.07%	−18.3658

**Figure 2:**
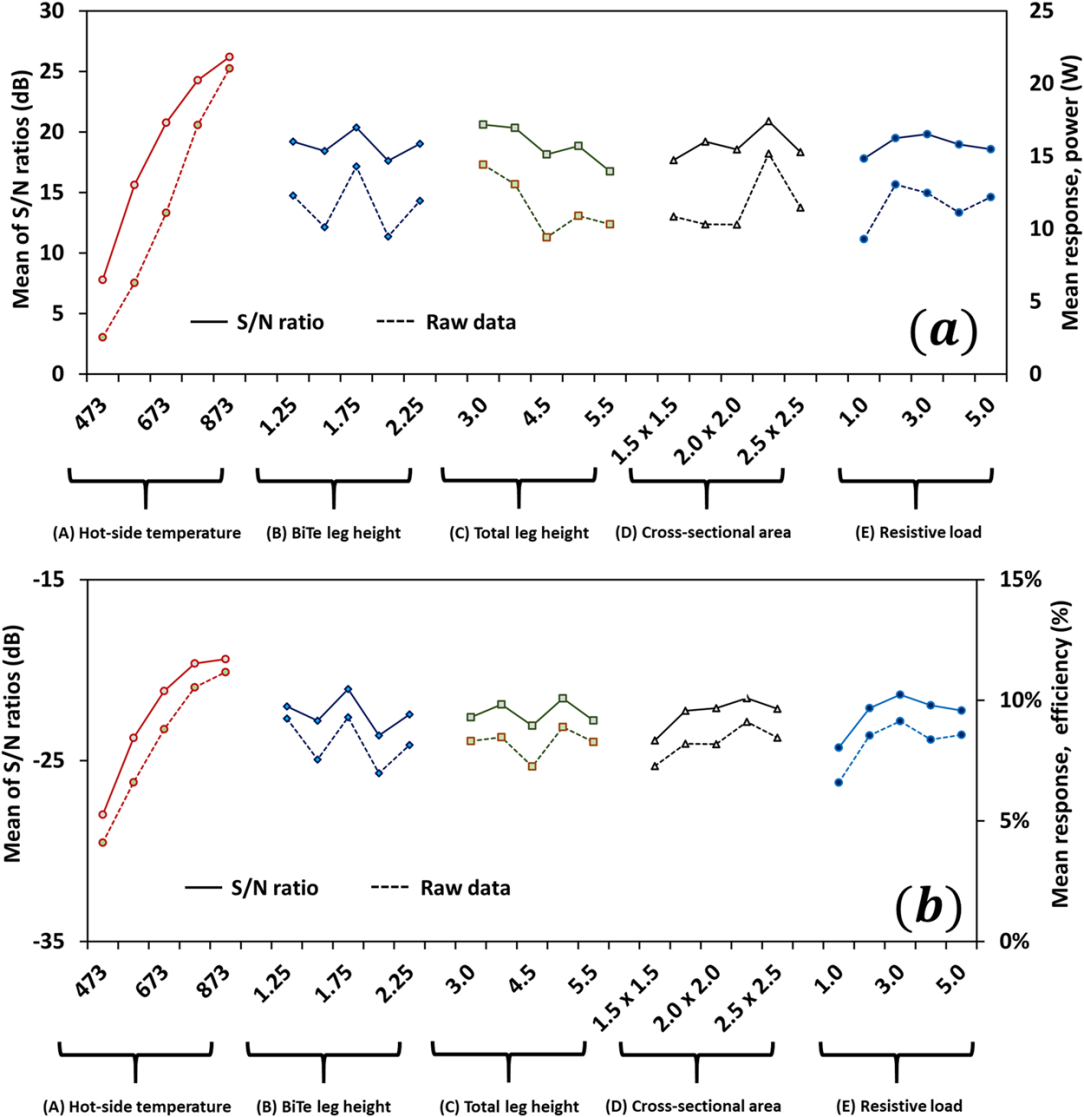
Taguchi optimization. (**a**) Mean of raw data (dotted line) and mean of S/N ratios (solid line) for power output at different levels of the control factors. Combination A_5_B_3_C_1_D_4_E_3_ (hot-side temperature: 873 K, BiTe leg height: 1.75 mm, total leg height: 3.0 mm, cross-section area: 2.25 × 2.25 mm^2^, and resistive load: 3.0 Ω) has appeared as the optimal control factor setting for the highest power output. (**b**) Mean of raw data (dotted line) and mean of S/N ratios (solid line) for efficiency at various levels of the control factors. Combination A_5_B_3_C_4_D_4_E_3_ (hot-side temperature: 873 K, BiTe leg height: 1.75 mm, total leg height: 4.5 mm, cross-section area: 2.25 × 2.25 mm^2^, and resistive load: 3.0 Ω) is the optimal setting for the highest efficiency.

In the first stage of optimization, we have considered an ideal condition, i.e. effect of environmental noise factors are neglected by perfectly insulating side-walls of all p-n legs. Figure 2(a,b) show the mean response for the raw data and S/N data for power and efficiency. The mean response signifies an average value of the output response for each factor at various levels. For instance, the mean response of the raw data for power for parameter A (hot-side temperature) at level 1 (473 K) implies average of all the values of power for parameter A at level 1 in column 7 of Table 2. Likewise, the mean responses of raw data and S/N data for all other factors at various levels are calculated and shown in Fig. 2(a,b). Higher S/N ratio indicates larger effect of a control factor over noise; therefore, the level of a control that has highest S/N ratio is considered the optimal level for that control factor, and the combination of all optimal levels establishes the optimal setting for higher output. It can be seen from Fig. 2 that the combination A_5_B_3_C_1_D_4_E_3_ (hot-side temperature: 873 K, BiTe leg height: 1.75 mm, total leg height: 3.0 mm, cross-section area: 2.25 × 2.25 mm^2^, and resistive load: 3.0 Ω) has appeared as the optimal control factor setting for the highest power output. Similarly, combination A_5_B_3_C_4_D_4_E_3_ (hot-side temperature: 873 K, BiTe leg height: 1.75 mm, total leg height: 4.5 mm, cross-section area: 2.25 × 2.25 mm^2^, and resistive load: 3.0 Ω) is the optimal setting for the highest efficiency.

The optimal control factor setting can be used to predict the optimal output, Y, using equation (2)^67^:

2 $${Y}_{predicted}=\overline{X}+\sum _{i=1}^{m}(\overline{{X}_{i}}-\overline{X})$$

where $\overline{{X}_{i}}$ denotes the mean of the output results at the optimal level of factor *i*, $\overline{X}$ denotes the grand mean of all the output data, and *m* represents the total number of control factors. Using equation (2), the predicted values of highest power output and highest efficiency were found to be 30.93 W and 14.6%, respectively. The confirmation run conducted at A_5_B_3_C_1_D_4_E_3_ (hot-side temperature: 873 K, BiTe leg height: 1.75 mm, total leg height: 3.0 mm, cross-section area: 2.25 × 2.25 mm^2^, and resistive load: 3.0 Ω) provides the power output of 35.02 W, which differs by 11.7% from the predicted value. Similarly, the confirmation run conducted at combination A_5_B_3_C_4_D_4_E_3_ (hot-side temperature: 873 K, BiTe leg height: 1.75 mm, total leg height: 4.5 mm, cross-section area: 2.25 × 2.25 mm^2^, and resistive load: 3.0 Ω) shows the efficiency of 14.7%, which differs by less than 1.0% from the predicted value.

Tables 3 and 4 show the ANOVA table for power output and efficiency, respectively. It can be noted that the percentage contribution of hot-side temperature, BiTe leg height, total leg height, cross-section area, and resistive load on power output is 89.52%, 1.68%, 4.15%, 2.45%, and 1.02%, respectively. This implies that hot-side temperature is the most prominent factor that affects power output. The total leg height is the most prominent geometric parameter followed by cross-sectional area and BiTe leg height. The contribution from error term is 1.19%, indicating minor effect from the external factors not included in this study. Similarly, we note that the contribution of hot-side temperature, BiTe leg height, total leg height, cross-section area, and resistive load on efficiency is 76.16%, 5.38%, 2.35%, 4.59%, and 7.37%, respectively. Again, hot-side temperature is the most prominent factor that affects efficiency followed by resistive load. However, unlike power, BiTe leg height has appeared as the important geometric parameter for efficiency, followed by cross-sectional area and total leg height. Error term also has some small effect (4.15%).

**Table 3:** ANOVA table highlighting percentage contribution from various factors on output power.

Source of variation	Degree of freedom (DOF)	Sum of squares (SS)	Variance (V)	F-value (F)	Percentage contribution
(A) Hot-side temperature	4.00	1098.79	274.70	75.49	89.52%
(B) BiTe leg height	4.00	20.57	5.14	1.41	1.68%
(C) Total leg height	4.00	50.92	12.73	3.50	4.15%
(D) Cross-sectional area	4.00	30.11	7.53	2.07	2.45%
(E) Resistive load	4.00	12.46	3.12	0.86	1.02%
Error	4.00	14.55	3.64		1.19%
Total	24.00	1227.40

**Table 4:** ANOVA table highlighting percentage contribution from various factors on efficiency.

Source of variation	Degree of freedom (DOF)	Sum of squares (SS)	Variance (V)	F-value (F)	Percentage contribution
(A) Hot-side temperature	4.00	256.47	64.12	18.37	76.16%
(B) BiTe leg height	4.00	18.11	4.53	1.30	5.38%
(C) Total leg height	4.00	7.93	1.98	0.57	2.35%
(D) Cross-sectional area	4.00	15.46	3.87	1.11	4.59%
(E) Resistive load	4.00	24.82	6.20	1.78	7.37%
Error	4.00	13.96	3.49		4.15%
Total	24.00	336.74			

Taguchi results can be validated by comparing the results obtained using conventional full factorial optimization method. Full factorial design of optimization requires varying each control factors one-by-one at their various levels. For five parameters, each having five levels requires 5^5^ = 3125 optimization runs for a single trial of experimentation. We conducted 3125 simulations on the segmented TEG considering the ideal condition, i.e. effect of environmental noise factors are neglected by perfectly insulating side-walls of all p-n legs. Figures S1–S5 in the supplementary document show the power output as a function of resistive load at different legs dimensions and hot-side temperature. It can be observed that power output increases with increase in hot-side temperature. The effect of BiTe legs, i.e. segmentation, is high when total leg height is small. The optimal resistance changes with change in geometric dimension of p-n legs. Broadly, optimal resistance is large when length and width of p-n legs are small and vice-versa. The power output increases with decrease in total p-n legs height. The maximum power of 39.73 W occurs at BiTe leg height of 1.25 mm, total leg height of 3 mm, leg cross-sectional area of 2.25 × 2.25 mm^2^ and resistive load of 2.0 Ω at hot-side temperature of 873 K. Previously, we noted that Taguchi method predicted optimal setting for power output as A_5_B_3_C_1_D_4_E_3_ (hot-side temperature: 873 K, BiTe leg height: 1.75 mm, total leg height: 3.0 mm, cross-section area: 2.25 × 2.25 mm^2^, and resistive load: 3.0 Ω), where optimal power output was found to be 35.02 W. Therefore, the difference between actual optima and Taguchi optima is about 11.8%. Likewise, Figures S6–S10 in the supplementary document show the efficiency as a function of resistive load at different legs dimensions with hot-side temperature. It can be seen that maximum efficiency of 15.3% occurs at BiTe leg height of 1.5 mm, total leg height of 5 mm, legs cross-sectional area of 2.5 × 2.5 mm^2^ and resistive load of 2.0 Ω at hot-side temperature of 873 K. Taguchi method predicted optimal efficiency of 14.7% at setting A_5_B_3_C_4_D_4_E_3_ (hot-side temperature: 873 K, BiTe leg height: 1.75 mm, total leg height: 4.5 mm, cross-section area: 2.25 × 2.25 mm^2^, and resistive load: 3.0 Ω). Therefore, the difference between actual optima and Taguchi optima is about 3.8%. It can be concluded that Taguchi optimization method effectively predicts near-optimal geometric parameters and operating conditions of a segmented TEG with only 25 experimental runs against 3125 experiments needed by conventional full factorial optimization method.

**Figure 3:**
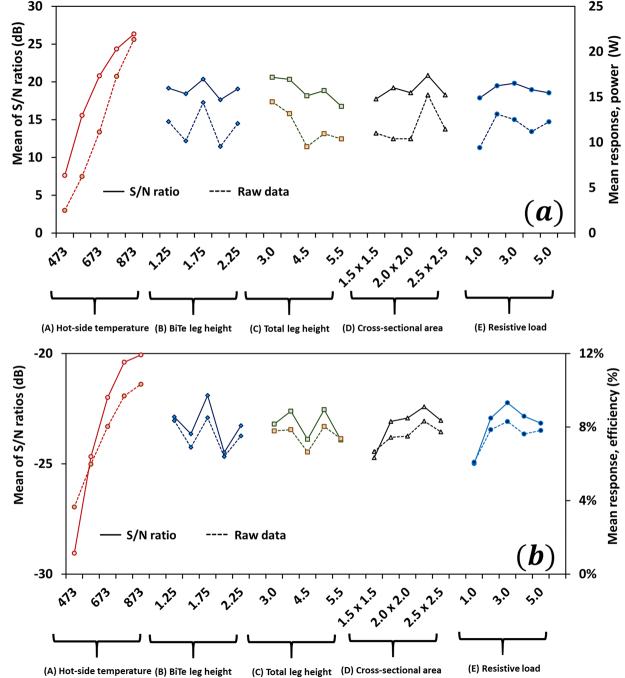
Effect of environmental factors on optimization of segmented TEGs. (**a**) Means of raw data (dotted line) and S/N ratios (solid line) for power at different levels of the control factors. (**b**) Means of raw data (dotted line) and S/N ratios (solid line) for efficiency at various levels of the control factors. The optimal control factors A_5_B_3_C_1_D_4_E_3_ (hot-side temperature: 873 K, BiTe leg height: 1.75 mm, total leg height: 3.0 mm, cross-section area: 2.25 × 2.25 mm^2^, and resistive load: 3.0 Ω) for power and A_5_B_3_C_4_D_4_E_3_ (hot-side temperature: 873 K, BiTe leg height: 1.75 mm, total leg height: 4.5 mm, cross-section area: 2.25 × 2.25 mm^2^, and resistive load: 3.0 Ω) for efficiency are same as those obtained for the ideal case, indicating the environment factors have no substantial effect on the optimization of segmented TEGs.

As of now, we have considered an ideal operating condition, where effect of noise factors is neglected. In practice, however, the performance of a segmented TEG is greatly affected by radiation and convection heat losses. We identify ambient temperature, ${T}_{\infty }$, and the total cooling coefficient, ${h}_{\infty }$, as the two main noise factors. Table S2 in Supplementary document shows power output obtained at seven different environment conditions. Trial 1 is performed at T_∞_ = 295 K and h = 0 W/m^2^-K. Trial 2, 3, and 4 are carried out at a fixed cooling coefficient h = 10 W/m^2^-K but at different ambient temperature T_∞_ = 285 K, 295 K, and 305 K, respectively. Trial 5, 6, and 7 have fixed ambient temperature T_∞_ = 295 K but different cooling coefficient h = 5 W/m^2^-K, h = 15 W/m^2^-K, and h = 20 W/m^2^-K, respectively. Table S3 in Supplementary document shows efficiency obtained at different environmental conditions as described above. Figure 3(a,b) show the mean response for the raw data and S/N data for power output and efficiency. It can be noted that the optimal setting for power output is A_5_B_3_C_1_D_4_E_3_ (hot-side temperature: 873 K, BiTe leg height: 1.75 mm, total leg height: 3.0 mm, cross-section area: 2.25 × 2.25 mm^2^, and resistive load: 3.0 Ω) and efficiency is A_5_B_3_C_4_D_4_E_3_ (hot-side temperature: 873 K, BiTe leg height: 1.75 mm, total leg height: 4.5 mm, cross-section area: 2.25 × 2.25 mm^2^, and resistive load: 3.0 Ω). These conditions are exactly same as those obtained for the ideal condition. This implies that the environment factors do not significantly affect the optimal configuration of segmented TEG.

**Table 5:** Normalized power output at seven different trials: trial 1 at T_∞_ = 295 K and h = 0 W/m^2^-K; trial 2 at T_∞_ = 285 K and h = 10 W/m^2^-K; trial 3 at T_∞_ = 295 K and h = 10 W/m^2^-K; trial 4 at T_∞_ = 305 K and h = 10 W/m^2^-K; trial 5 at T_∞_ = 295 K and h = 5 W/m^2^-K; trial 6 at T_∞_ = 295 K and h = 15 W/m^2^-K; trial 7 at T_∞_ = 295 K and h = 20 W/m^2^-K.

Control factors					Normalized power output							S/N (dB)
A	B	C	D	E	Trial 1	Trial 2	Trial 3	Trial 4	Trial 5	Trial 6	Trial 7	
1	1	1	1	1	0.06	0.06	0.06	0.06	0.06	0.06	0.06	−24.071
1	2	2	2	2	0.10	0.09	0.09	0.09	0.09	0.09	0.09	−20.612
1	3	3	3	3	0.08	0.08	0.08	0.08	0.08	0.08	0.08	−22.194
1	4	4	4	4	0.08	0.08	0.08	0.08	0.08	0.08	0.08	−22.229
1	5	5	5	5	0.04	0.04	0.04	0.04	0.04	0.04	0.04	−27.670
2	1	2	3	4	0.18	0.17	0.17	0.17	0.17	0.17	0.17	−15.323
2	2	3	4	5	0.17	0.17	0.17	0.17	0.17	0.17	0.16	−15.494
2	3	4	5	1	0.19	0.18	0.18	0.18	0.18	0.18	0.18	−14.793
2	4	5	1	2	0.11	0.11	0.11	0.11	0.11	0.10	0.10	−19.532
2	5	1	2	3	0.26	0.25	0.25	0.25	0.26	0.25	0.25	−11.877
3	1	3	5	2	0.31	0.31	0.31	0.31	0.31	0.30	0.30	−10.268
3	2	4	1	3	0.24	0.24	0.24	0.24	0.24	0.24	0.24	−12.309
3	3	5	2	4	0.29	0.28	0.28	0.28	0.28	0.28	0.28	−10.967
3	4	1	3	5	0.34	0.33	0.33	0.33	0.33	0.33	0.33	−9.5514
3	5	2	4	1	0.41	0.41	0.41	0.41	0.41	0.41	0.41	−7.7595
4	1	4	2	5	0.47	0.47	0.47	0.47	0.47	0.47	0.47	−6.5594
4	2	5	3	1	0.31	0.31	0.31	0.31	0.31	0.31	0.31	−10.176
4	3	1	4	2	0.78	0.77	0.77	0.77	0.77	0.77	0.77	−2.2570
4	4	2	5	3	0.47	0.46	0.47	0.47	0.47	0.46	0.46	−6.6426
4	5	3	1	4	0.42	0.43	0.43	0.43	0.42	0.43	0.43	−7.4345
5	1	5	4	3	0.73	0.73	0.73	0.73	0.72	0.73	0.73	−2.7689
5	2	1	5	4	0.63	0.62	0.62	0.62	0.63	0.62	0.62	−4.0948
5	3	2	1	5	0.71	0.72	0.72	0.72	0.72	0.72	0.73	−2.8455
5	4	3	2	1	0.36	0.37	0.37	0.37	0.37	0.37	0.37	−8.6881
5	5	4	3	2	0.57	0.58	0.58	0.58	0.58	0.58	0.58	−4.7786

**Table 6:** Normalized efficiency at seven different trials: trial 1 at T_∞_ = 295 K and h = 0 W/m^2^-K; trial 2 at T_∞_ = 285 K and h = 10 W/m^2^-K; trial 3 at T_∞_ = 295 K and h = 10 W/m^2^-K; trial 4 at T_∞_ = 305 K and h = 10 W/m^2^-K; trial 5 at T_∞_ = 295 K and h = 5 W/m^2^-K; trial 6 at T_∞_ = 295 K and h = 15 W/m^2^-K; trial 7 at T_∞_ = 295 K and h = 20 W/m^2^-K.

Control factors					Normalized efficiency							S/N (dB)
A	B	C	D	E	Trial 1	Trial 2	Trial 3	Trial 4	Trial 5	Trial 6	Trial 7	
1	1	1	1	1	0.18	0.20	0.20	0.20	0.20	0.20	0.20	−14.099
1	2	2	2	2	0.32	0.35	0.35	0.35	0.36	0.34	0.34	−9.2853
1	3	3	3	3	0.34	0.36	0.37	0.37	0.38	0.36	0.35	−8.8572
1	4	4	4	4	0.32	0.33	0.33	0.34	0.35	0.32	0.31	−9.7271
1	5	5	5	5	0.23	0.24	0.24	0.24	0.25	0.23	0.22	−12.588
2	1	2	3	4	0.46	0.50	0.50	0.51	0.52	0.49	0.49	−6.1112
2	2	3	4	5	0.42	0.45	0.45	0.46	0.47	0.44	0.43	−7.0113
2	3	4	5	1	0.52	0.56	0.57	0.57	0.58	0.55	0.54	−5.0984
2	4	5	1	2	0.32	0.34	0.34	0.34	0.35	0.33	0.32	−9.5673
2	5	1	2	3	0.52	0.58	0.58	0.58	0.59	0.57	0.57	−4.9065
3	1	3	5	2	0.65	0.71	0.71	0.71	0.73	0.70	0.68	−3.1452
3	2	4	1	3	0.51	0.55	0.55	0.56	0.57	0.54	0.53	−5.2996
3	3	5	2	4	0.68	0.72	0.72	0.73	0.75	0.70	0.68	−2.9569
3	4	1	3	5	0.59	0.66	0.67	0.67	0.67	0.66	0.65	−3.7296
3	5	2	4	1	0.56	0.63	0.63	0.63	0.64	0.63	0.63	−4.1780
4	1	4	2	5	0.85	0.91	0.92	0.92	0.94	0.89	0.87	−0.9127
4	2	5	3	1	0.57	0.62	0.62	0.62	0.64	0.61	0.60	−4.2900
4	3	1	4	2	0.79	0.90	0.90	0.90	0.91	0.90	0.90	−1.0609
4	4	2	5	3	0.73	0.81	0.81	0.81	0.82	0.80	0.79	−2.0007
4	5	3	1	4	0.64	0.71	0.72	0.72	0.73	0.70	0.69	−3.0957
5	1	5	4	3	1.00	1.08	1.08	1.08	1.11	1.06	1.04	0.5346
5	2	1	5	4	0.74	0.84	0.84	0.84	0.85	0.84	0.84	−1.6789
5	3	2	1	5	0.81	0.92	0.92	0.92	0.93	0.92	0.91	−0.8917
5	4	3	2	1	0.41	0.47	0.47	0.47	0.47	0.47	0.47	−6.7237
5	5	4	3	2	0.82	0.91	0.91	0.91	0.93	0.90	0.89	−0.9762

The segmented TEG has different optimal parameters for power and efficiency. As it can be noticed, at the optimized condition for power (A_5_B_3_C_1_D_4_E_3_), efficiency of segmented TEG is only 12.8% against 14.7% at A_5_B_3_C_4_D_4_E_3_, which is optimal condition for efficiency. Likewise, at the optimized condition for efficiency (A_5_B_3_C_4_D_4_E_3_), power of segmented TEG is only 28.1 W against 35.02 W at A_5_B_3_C_1_D_4_E_3_, which is optimal condition for power. In order to optimize both power and efficiency, we need to determine a combined S/N ratio. The combined “larger is better” S/N ratio for simultaneous optimization of two physical quantities can be calculated by

3 $${\rm{Larger}}\,{\rm{is}}\,{\rm{better}}\,S/N(dB)=-10\,\mathrm{log}[\frac{1}{r}\sum _{i=1}^{r}(\frac{w}{{y}_{i}^{2}}+\frac{1-w}{{z}_{i}^{2}})]$$

where y_i_ and z_i_ denote normalized data points for power and efficiency, respectively, and w is weight of power output.

**Figure 4:**
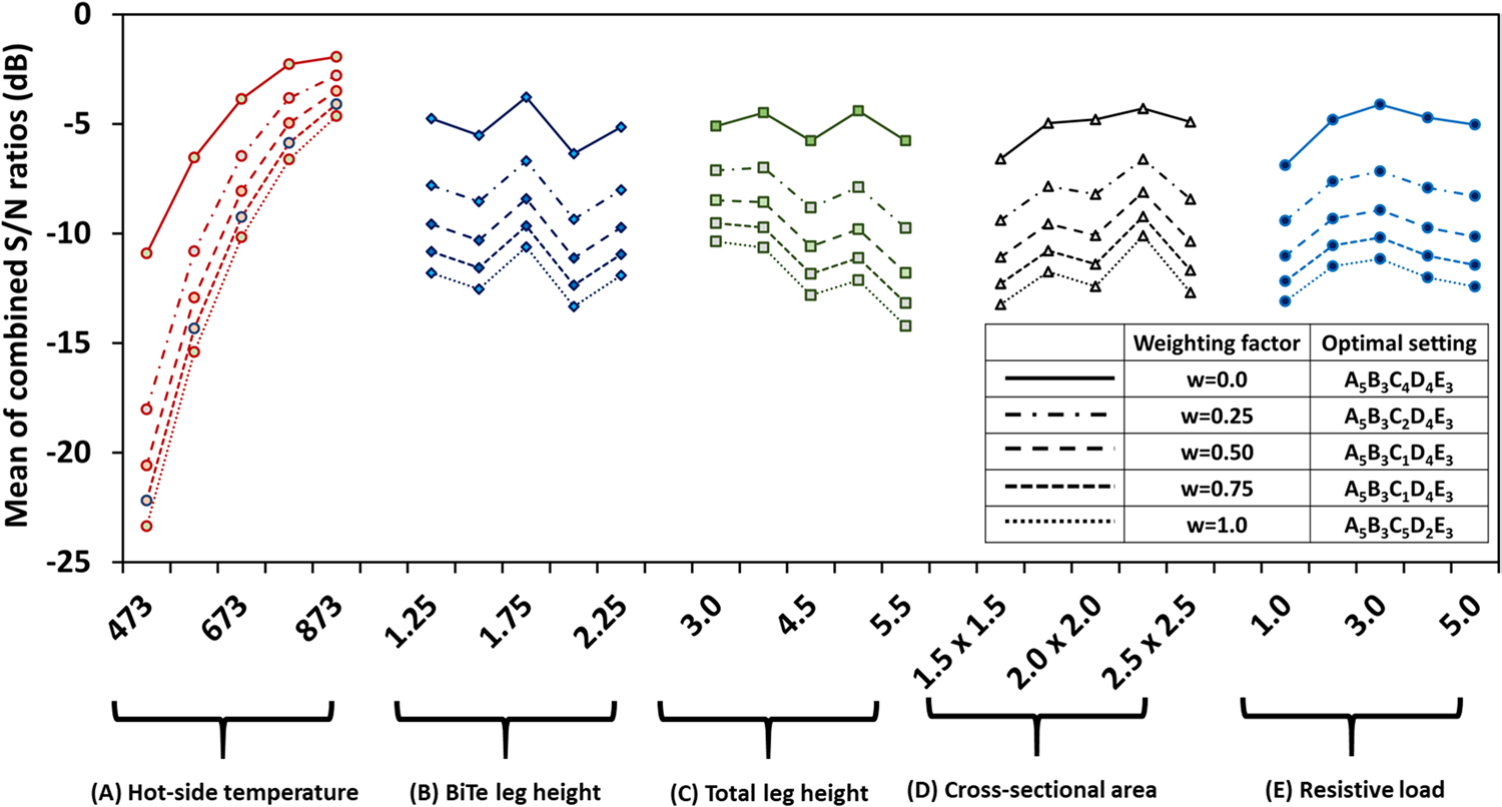
Combined S/N ratio for simultaneous optimization of power output and efficiency by segmented TEG. Weighting factor, w and the corresponding optimum setting are shown in the inset.

Tables 5 and 6 show the normalized values (with respect to their optimal value) for power and efficiency obtained at different operating conditions. The combined S/N ratios were calculated at different weighing factors using equation (3) and the values are plotted in Fig. 4. The value of weighting factor, w and the corresponding optimum setting are shown in the inset.

**Figure 5:**
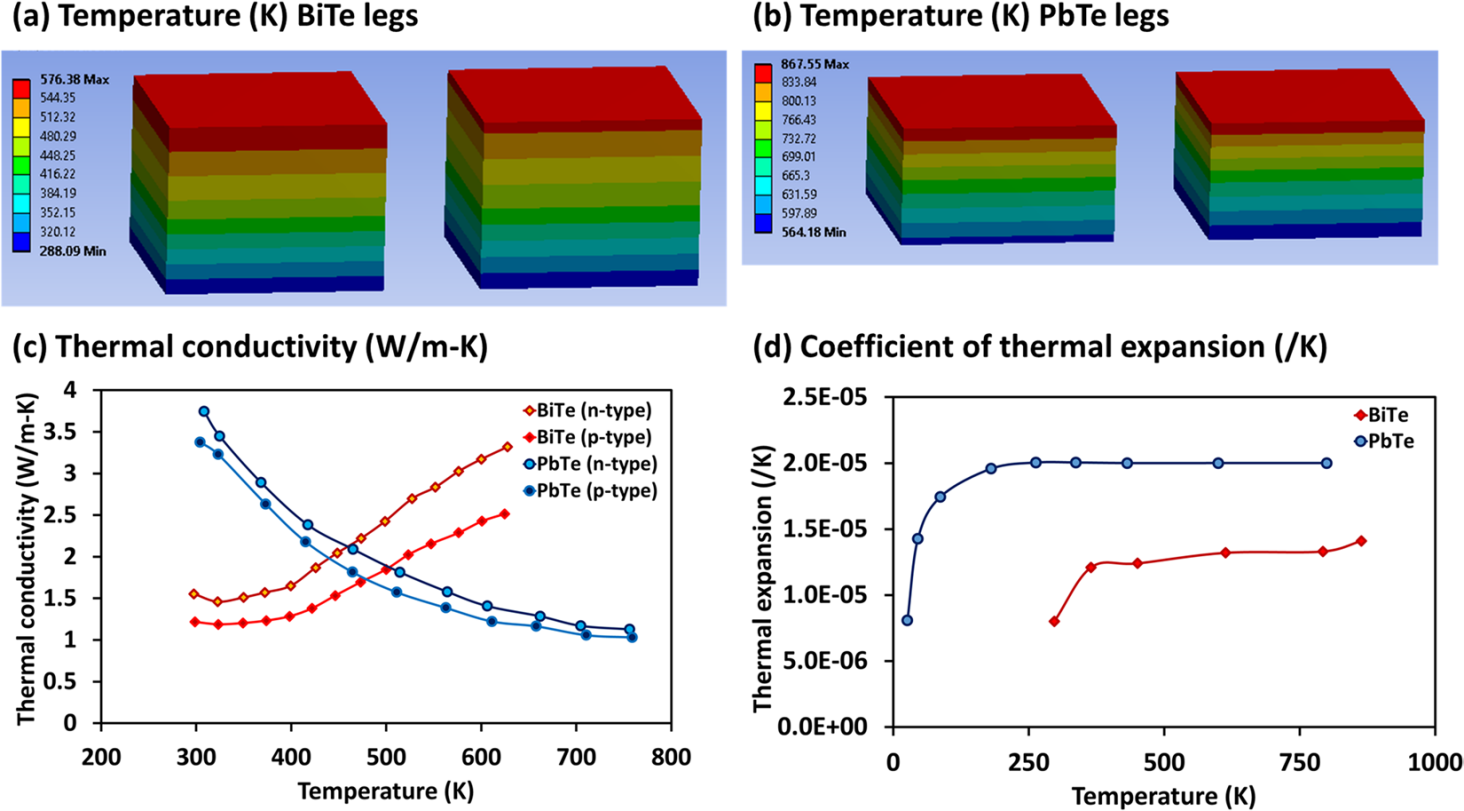
Temperature distribution in segmented TEG, when hot-side and cold-side are maintained at 873 K and 283 K, respectively. (**a**) Temperature distribution in BiTe leg. (**b**) Temperature distribution in PbTe leg. (**c**) Total thermal conductivity for p-type and n-type nanostructured BiTe and PbTe thermoelectric materials^15^. (**d**) Coefficient of thermal expansion for bulk BiTe and PbTe thermoelectric materials^69^. Difference in temperature gradients and mismatch in thermal expansion coefficients among various components of segmented TEGs may result in large thermal stresses in p- and n-type legs.

It should be noted that although conversion efficiency and output power are the two most important performance indicators of TEGs, their thermo-mechanical behavior cannot be ignored, especially at high temperature operation. Thermal stress is a major concern in the case of segmented TEGs as they are designed to operate under large temperature difference^68^. Figure 5(a,b) show the temperature distribution in BiTe and PbTe legs, when hot-side and cold-side are maintained at 873 K and 283 K, respectively. Figure 5(c) shows thermal conductivity of nanostructured BiTe and PbTe materials^15^. Figure 5(d) shows the coefficient of thermal expansion for bulk BiTe and PbTe materials^69^, as this information was not available in ref.^15^ for nanostructured materials. From this data, we can note the differences in thermo-mechanical properties of two thermoelectric materials. Difference in temperature gradients and mismatch in thermal expansion coefficients among various components of segmented TEGs may result in large thermal stresses in p- and n-type legs, which negatively influences the longevity of the TEG modules^70^. Prior studies have shown that concentration of thermal stresses is higher at the interfaces^71^. The difference in thermal conductivity between two materials stimulates high temperature gradient across the contact layer causing high thermal stress in the contact regions^70^. The maximum thermal stress level has been found to depend upon many factors including the hot-side temperature and the length of segments constituting p-type and n-type legs^72^. A proper design of segmented module will need to account for these stress concentrated zones by using diffusion barrier and matching interface layers.

## Conclusion

In summary, we employed numerical techniques along with Analysis of variance (ANOVA) and Taguchi optimization method to optimize segmented TEGs. Different design parameters, such as geometrical dimensions of p-n legs, height of segmentation, hot-side temperature, and load resistance, were considered to optimize power output and efficiency of segmented TEGs of size 40 × 40 mm^2^. Using the state-of-the-art TE materials and appropriate statistical tools, we accomplished near-optimum TEG configuration with only 25 experiments against 3125 experiments needed by the conventional optimization methods. The effect of environmental factors on the optimization of segmented TEGs was also studied. Lastly, Taguchi results were validated against the results obtained using traditional optimization technique and a TEG configuration for simultaneous optimization of power and efficiency was obtained. The major findings of the paper are summarized below:

- Under different levels of geometric parameters examined, Taguchi method showed that the segmented p-n legs having cross-sectional area of 2.25 × 2.25 mm^2^, BiTe leg height of 1.75 mm, and total leg height of 3.0 mm provided highest power output.
- Full factorial optimization method suggested optimized leg cross-sectional area of 2.25 × 2.25 mm^2^, BiTe height of 1.25 mm, and total leg height of 3 mm for highest power output.
- The difference between Taguchi results and full factorial optimization results for optimal power was found to be around 11.8%.
- Likewise, Taguchi method showed that the segmented p-n legs having cross-sectional area of 2.25 × 2.25 mm^2^, BiTe leg height of 1.75 mm, and total leg height of 4.5 mm provided highest efficiency.
- Full factorial optimization method suggested optimized leg cross-sectional area of 2.5 × 2.5 mm^2^, BiTe height of 1.5 mm, and total leg height of 5 mm for highest efficiency.
- The difference between Taguchi results and full factorial optimization results for optimal efficiency was found to be around 3.8%.
- Environmental factors such as ambient temperature and cooling coefficient were found to have insignificant effect on the optimization of segmented TEG modules.
- The optimal control factor setting for simultaneous optimization for power and efficiency were found to vary with change in the weighting factor.

## Methods

Mathematical formulation and numerical modeling: Thermoelectric effect is the resultant of Joule heating, Seebeck effect, Peltier effect, and Thomson effect. Modeling a TEG, therefore, requires coupling all these phenomena simultaneously. There are several analytical and numerical models in the literature^73–78^, which are mostly based on the principle of conservation of energy and continuity of electric charge. While some of the analytical and numerical models proposed in the literature are simplified one-dimensional cases^79,80^, few studies^73,81–83^ provide complex three-dimensional models for TE devices. Segmentation adds further complication in the numerical model because of the continuity requirements at the interfaces. The thermal and electrical contact resistances are very important factors that play crucial role in determining the overall performance of segmented TEGs but have been neglected in prior models^49,84,85^.

Using one-dimensional TEG model, the open circuit voltage (V_OC_) across the two terminals of a segmented thermocouple can be analytically obtained by integrating the Seebeck coefficients (α) of the constituent p-type and n-type pellets over the given temperature difference.

4 $${V}_{OC}=\sum _{i=1}^{{m}_{p}}\int {\alpha }_{i,p}(T)dT-\sum _{i=1}^{{m}_{n}}\int {\alpha }_{i,n}(T)dT$$

where subscript p and n denote p-type and n-type materials, α_i,p_ and α_i,n_ denote the temperature dependent Seebeck coefficient of i^th^ p-type and n-type pellet and m_p_ and m_n_ denote the number of p- and n-type pellets constituting one thermocouple. In order to simplify the calculation, an average Seebeck coefficients,$\overline{{\alpha }_{i,p}}$ and $\overline{{\alpha }_{i,n}}$, can be used, which are given as:

5 $$\overline{{\alpha }_{p}}=\frac{\sum _{i=1}^{{m}_{p}}\int {\alpha }_{i,p}(T)dT}{{T}_{h}-{T}_{c}}$$

6 $$\overline{{\alpha }_{n}}=\frac{\sum _{i=1}^{{m}_{n}}\int {\alpha }_{i,n}(T)dT}{{T}_{h}-{T}_{c}}$$

If N is total number of thermocouples used in a segmented TEG module, the open circuit voltage across the two terminals of the module can be given as:

7 $${V}_{m}=N\alpha ({T}_{h}-{T}_{c})$$

where $$\alpha =\overline{{\alpha }_{i,p}}-\overline{{\alpha }_{i,n}}$$

The power absorbed, P_h_, at the hot-side and the power released, P_c_, at the cold-side of the TEG module operating in steady state condition are expressed as^86,87^:

8 $${P}_{h}=N[I{T}_{h}\alpha -\frac{1}{2}{I}^{2}R+K({T}_{h}-{T}_{c})]$$

9 $${P}_{c}=N[I{T}_{c}\alpha +\frac{1}{2}{I}^{2}R+K({T}_{h}-{T}_{c})]$$

where R denotes the collective internal electrical resistance and K denotes the collective internal thermal conductance of an individual thermocouple, expressed as:

10 $$R=\sum _{i=1}^{{m}_{p}}(\frac{{\rho }_{p,i}{L}_{p,i}}{{A}_{p}})+\sum _{i=1}^{{m}_{n}}(\frac{{\rho }_{n,i}{L}_{n,i}}{{A}_{n}})$$

11 $$K=\frac{{A}_{p}}{\sum _{i=1}^{{m}_{p}}(\frac{{L}_{p,i}}{{k}_{p,i}})}+\frac{{A}_{n}}{\sum _{i=1}^{{m}_{n}}(\frac{{L}_{n,i}}{{k}_{n,i}})}$$

Here A_p_ and A_n_ denote cross-sectional area of p-type and n-type legs, L_i_ represents the length of i^th^ pellet, ρ_p,i_ and ρ_n,i_ represent electrical resistivity and k_p,i_ and k_n,i_ denote thermal conductivity of p-type and n-type thermoelectric materials.

When the segmented TEG is connected to an external resistive load, R_L_, the electric current, I, through the electric circuit is given as:

12 $${I}_{m}=\frac{{V}_{m}}{{R}_{total}}=\frac{N\alpha ({T}_{h}-{T}_{c})}{R+{R}_{L}}$$

The output power of the segmented TEG is calculated using:

13 $${P}_{out}={P}_{h}-{P}_{c}={I}^{2}R$$

The thermal-to-electrical energy conversion efficiency of the segmented TEG is then calculated as:

14 $$\eta =\frac{{P}_{out}}{{P}_{h}}$$

Using equations (4–13), equation (14) can be written as^88^:

15 $$\eta ={\eta }_{c}\frac{\beta }{(1+\beta )+{(1+\beta )}^{2}{(Z{T}_{h})}^{-1}-{\eta }_{c}/2}$$

where Carnot efficiency, ${\eta }_{c}=\frac{{T}_{h}-{T}_{c}}{{T}_{h}}$, $\beta =\frac{{R}_{L}}{R}$, and $Z=\frac{{\alpha }^{2}}{RK}$

In order to maximize efficiency, $\frac{d\eta }{d\beta }=0$. It can be shown that the maximum efficiency occurs at ${\beta }_{opt}=\sqrt{1+Z\overline{T}}$, where $\overline{T}=\frac{{T}_{h}+{T}_{c}}{2}$. Therefore, the maximum thermal-to-electrical energy conversion efficiency of an ideal segmented TEG (no contact resistances or thermal losses) operating under the optimal condition *β*_*opt*_ is given as^4^:

16 $${\eta }_{\max }=\frac{{\rm{\Delta }}T}{{T}_{h}}(\frac{\sqrt{1+Z\overline{T}}-1}{\sqrt{1+Z\overline{T}}+1-\frac{{\rm{\Delta }}T}{{T}_{h}}})$$

where $\Delta T$ is the temperature difference between hot-side and cold-side.

The one-dimensional analytical model derived above ignores contact resistances. In order to account for the thermal and electrical contact resistances, an improved one-dimensional theoretical model has been proposed^89^. The output voltage ${V}_{m}$ and current ${I}_{m}$, when the TEG module is operated at the matched load condition, are given by^89^:

17 $${V}_{m}=\frac{N\alpha ({T}_{h}-{T}_{c})}{1+2\varepsilon {l}_{c}\,/l}$$

18 $${I}_{m}=\frac{\alpha A({T}_{h}-{T}_{c})}{2\rho (\zeta +1)(1+2\varepsilon {l}_{c}/l)}$$

where A and *l* are the common cross-sectional area and length of the p- and n- legs, respectively, and *l*_*c*_ is the thickness of the contact layer. Further, $\zeta =\frac{2{\rho }_{c}}{\rho }$ and $\varepsilon =\frac{{\lambda }_{c}}{\lambda }$, where ρ and λ denote electrical and thermal resistivity of thermoelectric materials, where as ${\rho }_{c}$ and ${\lambda }_{c}$ denote electrical and thermal resistivity of contact layer.

The expressions for output power, P_out_, and efficiency η are now modified as^79^:

19 $${P}_{out}=\frac{{\alpha }^{2}}{2\rho }\frac{AN}{(\zeta +1){(1+2\varepsilon {l}_{c}/l)}^{2}}{({T}_{h}-{T}_{c})}^{2}$$

20 $$\eta =(\frac{{T}_{h}-{T}_{c}}{{T}_{h}}){({(1+2\varepsilon {l}_{c}/l)}^{2}[2-\frac{1}{2}(\frac{{T}_{h}-{T}_{c}}{{T}_{h}})+(\frac{4}{Z{T}_{h}})(\frac{1+\zeta }{1+2\varepsilon {l}_{c}})])}^{-1}$$

It can be noted that the one-dimensional models provide fairly accurate results when thermal gradient is small, material properties are constant with temperature, and there are small number of contact interfaces. In case of segmented TEGs, as the number of segmentations increases, the effect of contact resistances becomes more and more important. Moreover, the assumption for one-dimensionality deviates as thermal gradient is increased. The environmental effect, such as convective or radiative thermal losses, also needs to be taken into account for accurate results. Thus, we require a robust three-dimensional model to solve the coupled thermoelectric equations in steady state, which is given as^90^:

21 $$\mathop{q}\limits^{\rightharpoonup }=\pi \mathop{j}\limits^{\rightharpoonup }-\kappa \nabla T$$

22 $$\mathop{j}\limits^{\rightharpoonup }=\sigma (\mathop{E}\limits^{\rightharpoonup }-\alpha \nabla T)$$

where $\mathop{q}\limits^{\rightharpoonup }$, $\mathop{j}\limits^{\rightharpoonup }$, and $\mathop{E}\limits^{\rightharpoonup }$ stand for the heat flux vector, current density vector, and electric field intensity vector, respectively. α and π are Seebeck and Peltier coefficients, which are related as $\pi =T\alpha$. Equations21, 22 are usually solved using numerical methods. In this study, we employed a commercial finite element analysis (FEA) code, ANSYS v17.0 (ANSYS Inc., USA). ANSYS deduces the thermoelectric constitutive equations in the form of finite element matrix equation of thermoelectricity as^91^:

23 $$[\begin{array}{cc}[{C}^{t}] & 0\\ 0 & [{C}^{v}]\end{array}]\{\begin{array}{c}\{\dot{T}\}\\ \{\dot{V}\}\end{array}\}+[\begin{array}{cc}[{K}^{t}] & 0\\ \left[{K}^{vt}\right] & [{K}^{v}]\end{array}]\{\begin{array}{c}\{T\}\\ \{V\}\end{array}\}=\{\begin{array}{c}\{Q\}+\{{Q}^{P}\}\\ \{I\}\end{array}\}$$

where $[{C}^{t}]$ and $[{C}^{v}]$ are finite element specific heat matrix and dielectric permittivity coefficient matrix, respectively; $[{K}^{t}]$, $[{K}^{v}]$, and $[{K}^{vt}]$ are finite element thermal conductivity matrix, electrical conductivity coefficient matrix, and Seebeck coefficient coupling matrix, respectively; $\{Q\}$ denotes the sum of finite element heat generation load and convection surface heat flow vectors; $\{{Q}^{P}\}$ is finite element Peltier heat load vector; $\{T\}$, $\{V\}$, and $\{I\}$ are vectors of finite element nodal temperature, nodal electric potential, and nodal current, respectively.

## Electronic supplementary material

Supplementary information
