# Supplementary material for: Optimization of segmented thermoelectric generator using Taguchi and ANOVA techniques
Source: Sci Rep. 2017 Dec 1;7:16746. doi: 10.1038/s41598-017-16372-8 (PMC5711871; doi:10.1038/s41598-017-16372-8)
Supplement: Supplementary file 1 — Supplementary information [file 41598_2017_16372_MOESM1_ESM.doc]

**Supplementary information**

**Optimization of segmented thermoelectric generator using Taguchi and ANOVA techniques**

Ravi Anant Kishore ^a^, Mohan Sanghadasa ^b^ and Shashank Priya ^a^,*

^a^ Center for Energy Harvesting Materials and Systems (CEHMS), Virginia Tech, Blacksburg, VA 24061, USA.

^b^ Aviation and Missile Research, Development, and Engineering Center, US Army RDECOM, Redstone Arsenal, AL 35898, USA

**Taguchi** **optimization method**

Taguchi optimization method is a combination of mathematical and statistical techniques, which was originally developed by Genichi Taguchi ^66^ for the manufacturing industries to optimize the product quality and to minimize the production cost. However, this method is now widely used in diverse field of engineering ^50-59^. Taguchi method suggests a partial factorial design of optimization, where multiple process factors are varied simultaneously in a controlled fashion based on some standard orthogonal arrays. Orthogonal arrays identify the combination in which the process factors needs to be changed during the optimization process.

Table S1. L_25_ orthogonal array

| Control factors | | | | Output response | | | |
| --- | --- | --- | --- | --- | --- | --- | --- |
| Factor  (A) | Factor  (B) | Factor  (C) | Factor  (D) | Factor  (E) | Trial 1 | Trial 2 | Trial 3 |
| 1 | 1 | 1 | 1 | 1 | T_101_ | T_201_ | T_301_ |
| 1 | 2 | 2 | 2 | 2 | T_102_ | T_202_ | T_302_ |
| 1 | 3 | 3 | 3 | 3 | T_103_ | T_203_ | T_303_ |
| 1 | 4 | 4 | 4 | 4 | T_104_ | T_204_ | T_304_ |
| 1 | 5 | 5 | 5 | 5 | T_105_ | T_205_ | T_305_ |
| 2 | 1 | 2 | 3 | 4 | T_106_ | T_206_ | T_306_ |
| 2 | 2 | 3 | 4 | 5 | T_107_ | T_207_ | T_307_ |
| 2 | 3 | 4 | 5 | 1 | T_108_ | T_208_ | T_308_ |
| 2 | 4 | 5 | 1 | 2 | T_109_ | T_209_ | T_309_ |
| 2 | 5 | 1 | 2 | 3 | T_110_ | T_210_ | T_310_ |
| 3 | 1 | 3 | 5 | 2 | T_111_ | T_211_ | T_311_ |
| 3 | 2 | 4 | 1 | 3 | T_112_ | T_212_ | T_312_ |
| 3 | 3 | 5 | 2 | 4 | T_113_ | T_213_ | T_313_ |
| 3 | 4 | 1 | 3 | 5 | T_114_ | T_214_ | T_314_ |
| 3 | 5 | 2 | 4 | 1 | T_115_ | T_215_ | T_315_ |
| 4 | 1 | 4 | 2 | 5 | T_116_ | T_216_ | T_316_ |
| 4 | 2 | 5 | 3 | 1 | T_117_ | T_217_ | T_317_ |
| 4 | 3 | 1 | 4 | 2 | T_118_ | T_218_ | T_318_ |
| 4 | 4 | 2 | 5 | 3 | T_119_ | T_219_ | T_319_ |
| 4 | 5 | 3 | 1 | 4 | T_120_ | T_220_ | T_320_ |
| 5 | 1 | 5 | 4 | 3 | T_121_ | T_221_ | T_321_ |
| 5 | 2 | 1 | 5 | 4 | T_122_ | T_222_ | T_322_ |
| 5 | 3 | 2 | 1 | 5 | T_123_ | T_223_ | T_323_ |
| 5 | 4 | 3 | 2 | 1 | T_124_ | T_224_ | T_324_ |
| 5 | 5 | 4 | 3 | 2 | T_125_ | T_225_ | T_325_ |

**Orthogonal array (OA)**

In Taguchi optimization method, control factors are varied according to certain standard orthogonal arrays (OAs) such as L_4_, L_8_, L_9_, L_12_, L_16_, L_18_, and L_25_. Based on the number of process factors and their levels, an appropriate OA needs to be selected. In this study, we have considered five factors at each of the five levels, therefore L_25_ orthogonal array is chosen whose structure is shown in Table S1 in supplementary document. It can be noted that with five factors at five levels, the traditional optimization method requires 5^5^=3125 experiments, whereas, Taguchi method, per L_25_ orthogonal array, needs only 25 experiments to predict the optimal output.

**Signal-to-noise ratio**

Signal-to-noise (S/N) ratio is a very important concept in Taguchi optimization method. Signal measures the desirable component of the output response; whereas, the undesirable components is measured in terms of noise, which occurs due to variability in the process due to noise factors ^52,53,66^. Greater the value of S/N ratio, larger is the effect of control factors over the noise factors on the output response. Depending on the goal of the optimization, there are three methods to calculate S/N ratio: Larger is better, Smaller is better, and Nominal is best, which are calculated using equations below:

Larger is better (S1)

Smaller is better (S2)

Nominal is best $S/N(dB)=10\log\left[\frac{\left[\frac{1}{r}\left(\frac{T^{2}}{r}-\sigma_{r-1}^{2}\right)\right]}{\sigma_{r-1}^{2}}\right]$ (S3)

where *r* is the number of data points and *y_i_* is the value of *i^th^* data point. T denotes sum of all the data points and $\sigma_{r-1}^{2}$ denotes the variance.

$T=\sum_{i=1}^{r} y_{i}$ (S4)

$\sigma_{r-1}^{2}=\sum_{i=1}^{r} \frac{\left(y_{i}-\overline{y}\right)^{2}}{r-1}$ (S5)

$\overline{y}=\frac{\sum_{i=1}^{r} y_{i}}{r}$ (S6)

In this study, goal is to maximize the power output and the efficiency; therefore, larger is better concept for S/N ratio is used, which is calculated using equation (S1).

**Analysis of Variance (ANOVA)**

ANOVA stands for Analysis of Variance. It is a statistical technique used to test the relative significance of various control factors on the output response by comparing the variation in output caused by each factor relative to the total variation observed. ANOVA is performed by calculating three sets of parameters. First, we calculate the degrees of freedom (DOF)_i_, sum of squares (SS)_i_, variance V_i_, and contribution P_i_ for each control factor, *i*, using the equations below ^87^.

$\left(DOF\right)_{i}=k_{i}-1$ (S7)

$\left(SS\right)_{i}=\sum_{j=1}^{k_{i}} \frac{S_{ij}^{2}}{k_{i}}-S_{m}$ (S8)

where $k_{i}$ is the number of levels for the control factor *i*, $SS_{ij}$ is the sum of S/N ratios of factor *i* in level *j*, and S_m_ is a correction factor which is calculated using:

$S_{m}=\frac{1}{n}\left(\sum_{i=1}^{n} \left(\frac{S}{N}\right)_{i}\right)^{2}$ (S9)

$V_{i}=\frac{\left(SS\right)_{i}}{\left(DOF\right)_{i}}$ (S10)

$P_{i}=\frac{\left(SS\right)_{i}}{\left(SS\right)_{T}}$ (S11)

where n denotes the total number of experiments and $\left(SS\right)_{T}$ is called the total sum of squares and is calculated as:

$\left(SS\right)_{T}=\sum_{i=1}^{n} \left(\frac{S}{N}\right)_{i}^{2}-S_{m}$ (S12)

In the second step, we calculate the degrees of freedom (DOF)_e_, sum of squares (SS)_e_, variance V_e_, and contribution P_e_ for the error term, e, which signifies the collective effect of all the external factors not considered in the study, including experimental error and noise factors.

$\left(DOF\right)_{e}=\left(DOF\right)_{T}-\sum_{i=1}^{n} \left(DOF\right)_{i}$ (S13)

$\left(SS\right)_{e}=\left(SS\right)_{T}-\sum_{i=1}^{n} \left(SS\right)_{i}$ (S14)

$V_{e}=\frac{\left(SS\right)_{e}}{\left(DOF\right)_{e}}$ (S15)

$P_{e}=\frac{\left(SS\right)_{e}}{\left(SS\right)_{T}}$ (S16)

where $\left(DOF\right)_{T}$ denotes the total degree of freedom and is equal to n-1

In the third step, for every control factor, *i*, we compare the variance over the error variance to calculate a term called F-value.

$\left(F-value\right)_{i}=\frac{V_{i}}{V_{e}}$ (S17)

In this study, we have used a commercial program, Minitab 17 (Minitab Inc., USA), to obtain ANOVA and S/N ratios.

Table S2. Power output at seven different trials: trial 1 at T_∞_ = 295K and h = 0 W/m^2^-K; trial 2 at T_∞_ = 285 K and h = 10 W/m^2^-K; trial 3 at T_∞_ = 295 K and h = 10 W/m^2^-K; trial 4 at T_∞_ = 305 K and h = 10 W/m^2^-K; trial 5 at T_∞_ = 295 K and h = 5 W/m^2^-K; trial 6 at T_∞_ = 295 K and h =15 W/m^2^-K; trial 7 at T_∞_ = 295 K and h = 20 W/m^2^-K.

| Control factors | | | | | Power output (W) | | | | | | | S/N  (dB) |
| --- | --- | --- | --- | --- | --- | --- | --- | --- | --- | --- | --- | --- |
| A | B | C | D | E | Trial 1 | Trial 2 | Trial 3 | Trial 4 | Trial 5 | Trial 6 | Trial 7 |
| 1 | 1 | 1 | 1 | 1 | 2.24 | 2.21 | 2.21 | 2.22 | 2.23 | 2.20 | 2.19 | 6.9041 |
| 1 | 2 | 2 | 2 | 2 | 3.36 | 3.29 | 3.30 | 3.30 | 3.33 | 3.27 | 3.24 | 10.364 |
| 1 | 3 | 3 | 3 | 3 | 2.81 | 2.74 | 2.75 | 2.75 | 2.78 | 2.72 | 2.70 | 8.7820 |
| 1 | 4 | 4 | 4 | 4 | 2.80 | 2.73 | 2.74 | 2.74 | 2.77 | 2.71 | 2.68 | 8.7473 |
| 1 | 5 | 5 | 5 | 5 | 1.50 | 1.46 | 1.46 | 1.47 | 1.48 | 1.44 | 1.43 | 3.3061 |
| 2 | 1 | 2 | 3 | 4 | 6.13 | 6.06 | 6.06 | 6.07 | 6.10 | 6.03 | 5.99 | 15.653 |
| 2 | 2 | 3 | 4 | 5 | 6.02 | 5.94 | 5.94 | 5.95 | 5.98 | 5.91 | 5.87 | 15.481 |
| 2 | 3 | 4 | 5 | 1 | 6.50 | 6.44 | 6.44 | 6.45 | 6.47 | 6.41 | 6.38 | 16.182 |
| 2 | 4 | 5 | 1 | 2 | 3.73 | 3.74 | 3.74 | 3.74 | 3.74 | 3.73 | 3.72 | 11.443 |
| 2 | 5 | 1 | 2 | 3 | 9.00 | 9.02 | 9.01 | 9.01 | 9.01 | 9.02 | 9.03 | 19.098 |
| 3 | 1 | 3 | 5 | 2 | 10.91 | 10.84 | 10.84 | 10.85 | 10.88 | 10.82 | 10.79 | 20.707 |
| 3 | 2 | 4 | 1 | 3 | 8.50 | 8.59 | 8.58 | 8.57 | 8.52 | 8.62 | 8.65 | 18.665 |
| 3 | 3 | 5 | 2 | 4 | 9.99 | 10.02 | 10.02 | 10.02 | 9.99 | 10.02 | 10.00 | 20.008 |
| 3 | 4 | 1 | 3 | 5 | 11.76 | 11.79 | 11.78 | 11.78 | 11.78 | 11.79 | 11.79 | 21.423 |
| 3 | 5 | 2 | 4 | 1 | 14.34 | 14.49 | 14.49 | 14.48 | 14.42 | 14.54 | 14.60 | 23.215 |
| 4 | 1 | 4 | 2 | 5 | 16.58 | 16.61 | 16.61 | 16.61 | 16.61 | 16.64 | 16.71 | 24.415 |
| 4 | 2 | 5 | 3 | 1 | 10.74 | 11.00 | 10.99 | 10.98 | 10.89 | 11.03 | 11.10 | 20.798 |
| 4 | 3 | 1 | 4 | 2 | 27.19 | 27.29 | 27.29 | 27.28 | 27.24 | 27.33 | 27.36 | 28.718 |
| 4 | 4 | 2 | 5 | 3 | 16.48 | 16.46 | 16.46 | 16.46 | 16.48 | 16.46 | 16.47 | 24.332 |
| 4 | 5 | 3 | 1 | 4 | 14.74 | 15.09 | 15.08 | 15.06 | 14.95 | 15.14 | 15.18 | 23.540 |
| 5 | 1 | 5 | 4 | 3 | 25.56 | 25.70 | 25.68 | 25.66 | 25.52 | 25.91 | 26.03 | 28.205 |
| 5 | 2 | 1 | 5 | 4 | 21.93 | 22.09 | 22.08 | 22.08 | 22.01 | 22.15 | 22.22 | 26.880 |
| 5 | 3 | 2 | 1 | 5 | 25.03 | 25.50 | 25.48 | 25.46 | 25.22 | 25.75 | 26.04 | 28.129 |
| 5 | 4 | 3 | 2 | 1 | 12.61 | 13.06 | 13.04 | 13.03 | 12.86 | 13.18 | 13.32 | 22.285 |
| 5 | 5 | 4 | 3 | 2 | 20.07 | 20.41 | 20.40 | 20.38 | 20.30 | 20.54 | 20.76 | 26.195 |

Table S3. Efficiency at seven different trials: trial 1 at T_∞_ = 295K and h = 0 W/m^2^-K; trial 2 at T_∞_ = 285 K and h = 10 W/m^2^-K; trial 3 at T_∞_ = 295 K and h = 10 W/m^2^-K; trial 4 at T_∞_ =305 K and h = 10 W/m^2^-K; trial 5 at T_∞_ = 295 K and h = 5 W/m^2^-K; trial 6 at T_∞_ =295 K and h = 15 W/m^2^-K; trial 7 at T_∞_ = 295 K and h = 20 W/m^2^-K.

| Control factors | | | | | Efficiency | | | | | | | S/N  (dB) |
| --- | --- | --- | --- | --- | --- | --- | --- | --- | --- | --- | --- | --- |
| A | B | C | D | E | Trial 1 | Trial 2 | Trial 3 | Trial 4 | Trial 5 | Trial 6 | Trial 7 |
| 1 | 1 | 1 | 1 | 1 | 2.7% | 2.4% | 2.5% | 2.5% | 2.5% | 2.4% | 2.3% | -32.214 |
| 1 | 2 | 2 | 2 | 2 | 4.7% | 4.2% | 4.3% | 4.3% | 4.5% | 4.1% | 3.9% | -27.413 |
| 1 | 3 | 3 | 3 | 3 | 5.0% | 4.4% | 4.5% | 4.5% | 4.7% | 4.2% | 4.0% | -26.998 |
| 1 | 4 | 4 | 4 | 4 | 4.7% | 4.0% | 4.1% | 4.1% | 4.3% | 3.8% | 3.6% | -27.880 |
| 1 | 5 | 5 | 5 | 5 | 3.4% | 2.9% | 2.9% | 3.0% | 3.1% | 2.7% | 2.5% | -30.755 |
| 2 | 1 | 2 | 3 | 4 | 6.8% | 6.1% | 6.2% | 6.2% | 6.4% | 5.9% | 5.6% | -24.237 |
| 2 | 2 | 3 | 4 | 5 | 6.2% | 5.5% | 5.5% | 5.6% | 5.9% | 5.3% | 5.0% | -25.148 |
| 2 | 3 | 4 | 5 | 1 | 7.7% | 6.9% | 6.9% | 6.9% | 7.3% | 6.6% | 6.3% | -23.232 |
| 2 | 4 | 5 | 1 | 2 | 4.7% | 4.1% | 4.1% | 4.2% | 4.4% | 3.9% | 3.7% | -27.719 |
| 2 | 5 | 1 | 2 | 3 | 7.6% | 7.1% | 7.1% | 7.1% | 7.3% | 6.8% | 6.6% | -23.017 |
| 3 | 1 | 3 | 5 | 2 | 9.5% | 8.6% | 8.7% | 8.7% | 9.1% | 8.3% | 7.9% | -21.271 |
| 3 | 2 | 4 | 1 | 3 | 7.5% | 6.7% | 6.7% | 6.8% | 7.1% | 6.4% | 6.1% | -23.434 |
| 3 | 3 | 5 | 2 | 4 | 10.1% | 8.8% | 8.8% | 8.9% | 9.4% | 8.3% | 7.9% | -21.105 |
| 3 | 4 | 1 | 3 | 5 | 8.7% | 8.1% | 8.1% | 8.1% | 8.4% | 7.8% | 7.6% | -21.840 |
| 3 | 5 | 2 | 4 | 1 | 8.2% | 7.7% | 7.7% | 7.7% | 7.9% | 7.5% | 7.2% | -22.283 |
| 4 | 1 | 4 | 2 | 5 | 12.5% | 11.1% | 11.2% | 11.2% | 11.8% | 10.6% | 10.1% | -19.049 |
| 4 | 2 | 5 | 3 | 1 | 8.3% | 7.6% | 7.6% | 7.6% | 7.9% | 7.2% | 6.9% | -22.416 |
| 4 | 3 | 1 | 4 | 2 | 11.7% | 11.0% | 11.0% | 11.0% | 11.3% | 10.7% | 10.4% | -19.163 |
| 4 | 4 | 2 | 5 | 3 | 10.7% | 9.9% | 9.9% | 9.9% | 10.3% | 9.5% | 9.2% | -20.117 |
| 4 | 5 | 3 | 1 | 4 | 9.4% | 8.7% | 8.7% | 8.7% | 9.1% | 8.4% | 8.0% | -21.213 |
| 5 | 1 | 5 | 4 | 3 | 14.8% | 13.2% | 13.2% | 13.2% | 13.9% | 12.6% | 12.0% | -17.600 |
| 5 | 2 | 1 | 5 | 4 | 10.9% | 10.3% | 10.3% | 10.3% | 10.6% | 10.0% | 9.7% | -19.782 |
| 5 | 3 | 2 | 1 | 5 | 12.0% | 11.2% | 11.2% | 11.2% | 11.6% | 10.9% | 10.6% | -18.997 |
| 5 | 4 | 3 | 2 | 1 | 6.1% | 5.7% | 5.7% | 5.8% | 5.9% | 5.6% | 5.4% | -24.825 |
| 5 | 5 | 4 | 3 | 2 | 12.1% | 11.1% | 11.1% | 11.1% | 11.6% | 10.7% | 10.3% | -19.093 |


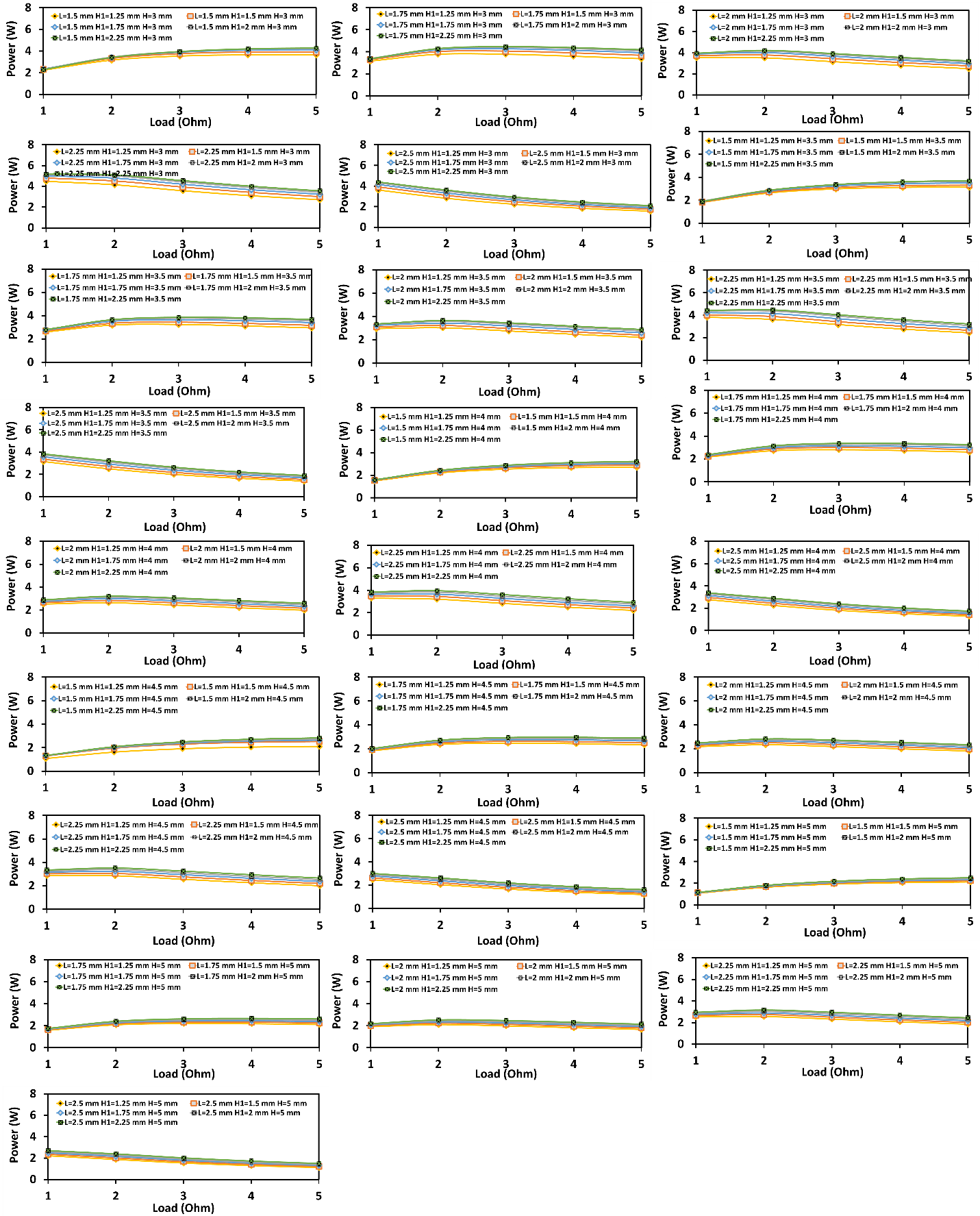


Figure S1. Power output vs. resistive load at different leg dimensions. Hot-side temperature, T_h_ = 473 K and cold-side temperature, T_c_ = 283 K.


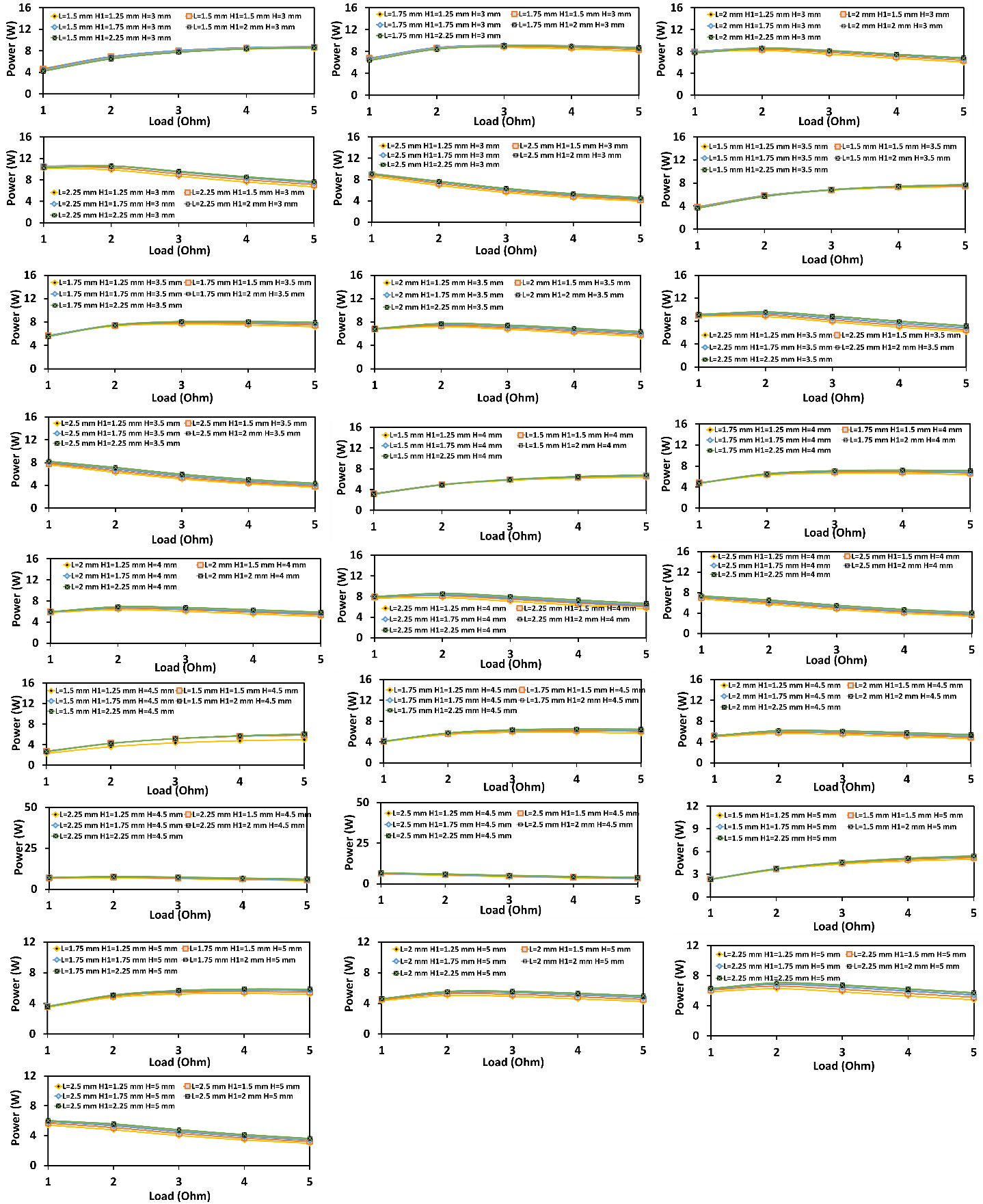


Figure S2. Power output vs. resistive load at different leg dimensions. Hot-side temperature, T_h_ = 573 K and cold-side temperature, T_c_ = 283 K.


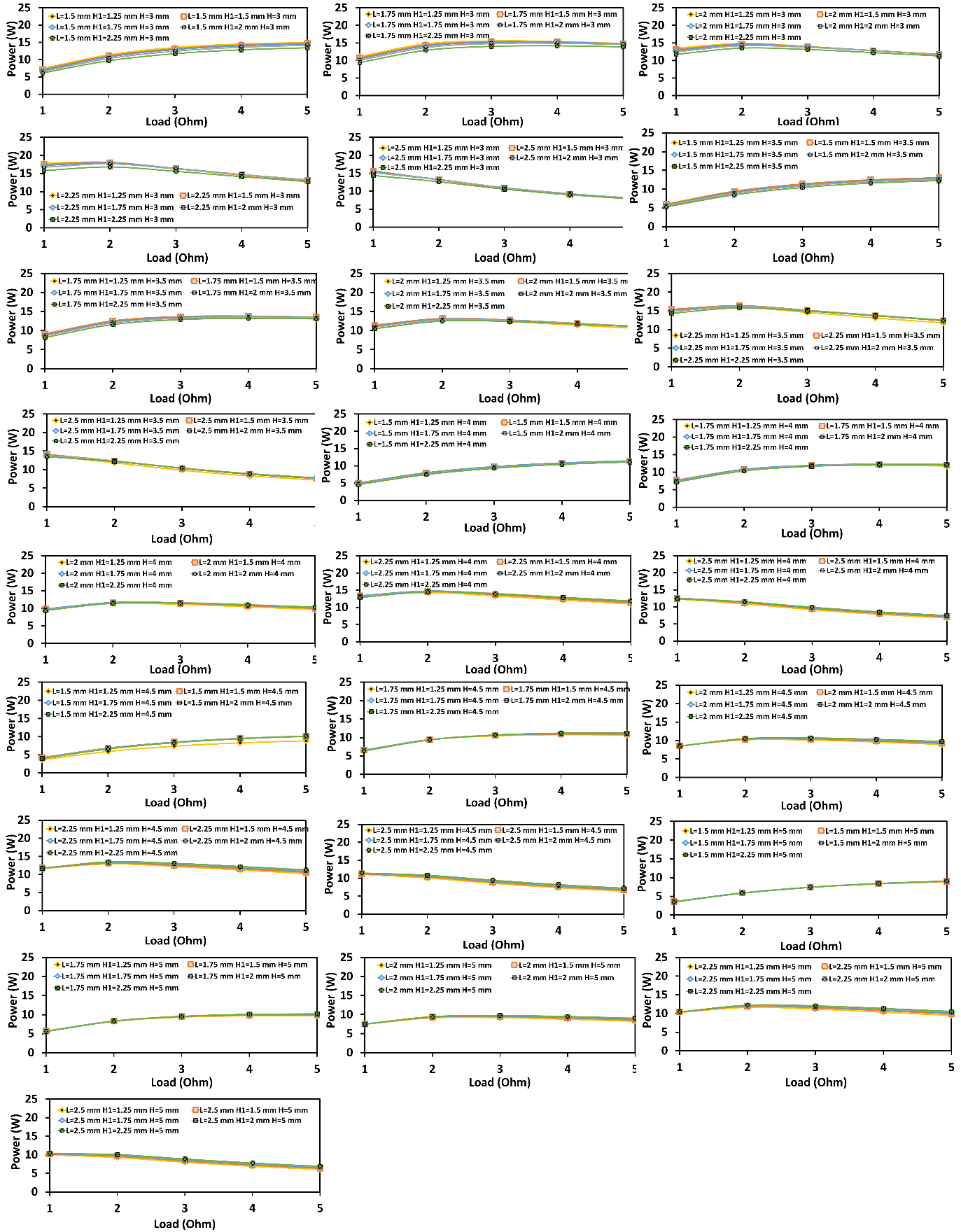


Figure S3. Power output vs. resistive load at different leg dimensions. Hot-side temperature, T_h_ = 673 K and cold-side temperature, T_c_ = 283 K.


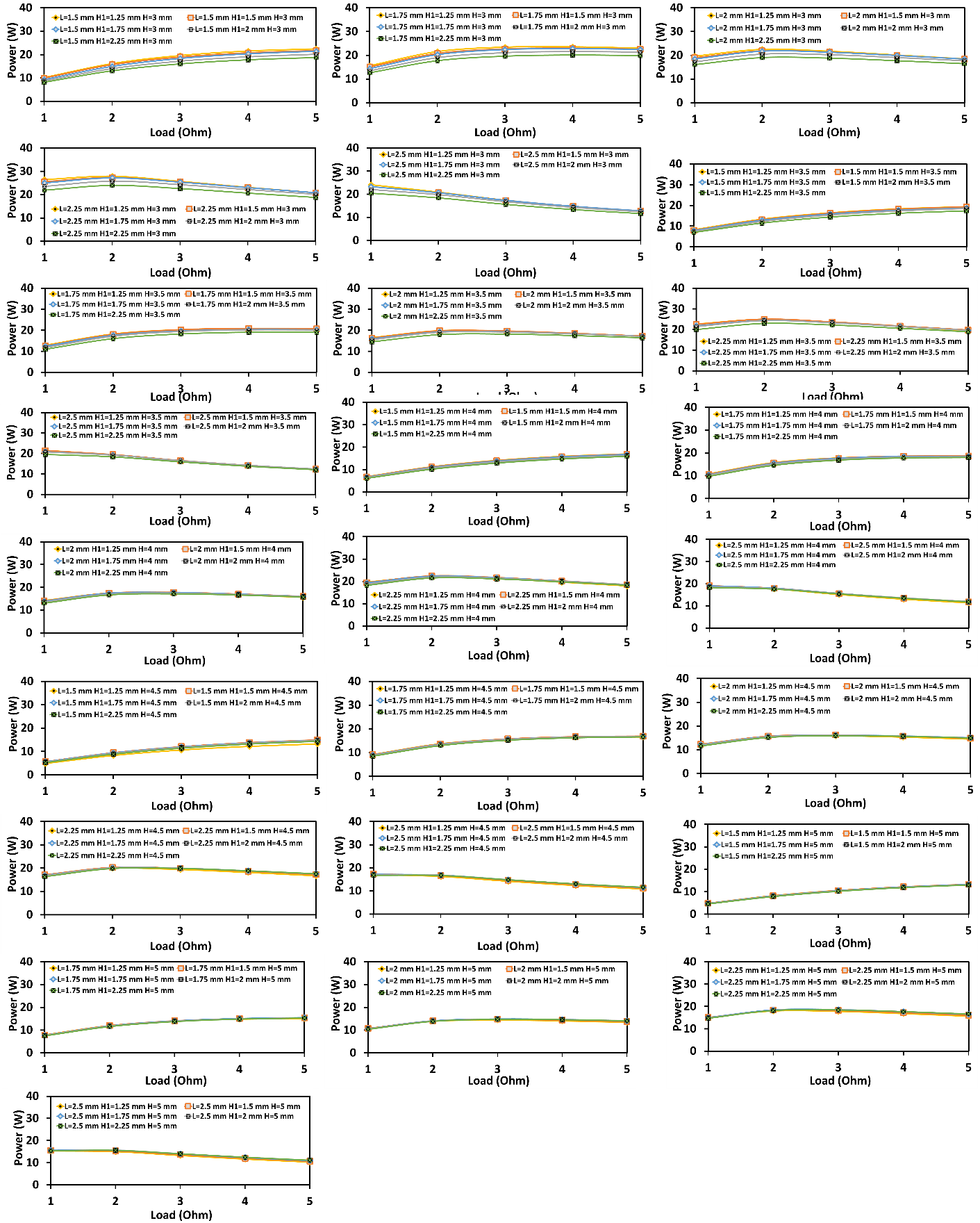


Figure S4. Power output vs. resistive load at different leg dimensions. Hot-side temperature, T_h_ = 773 K and cold-side temperature, T_c_ = 283 K.


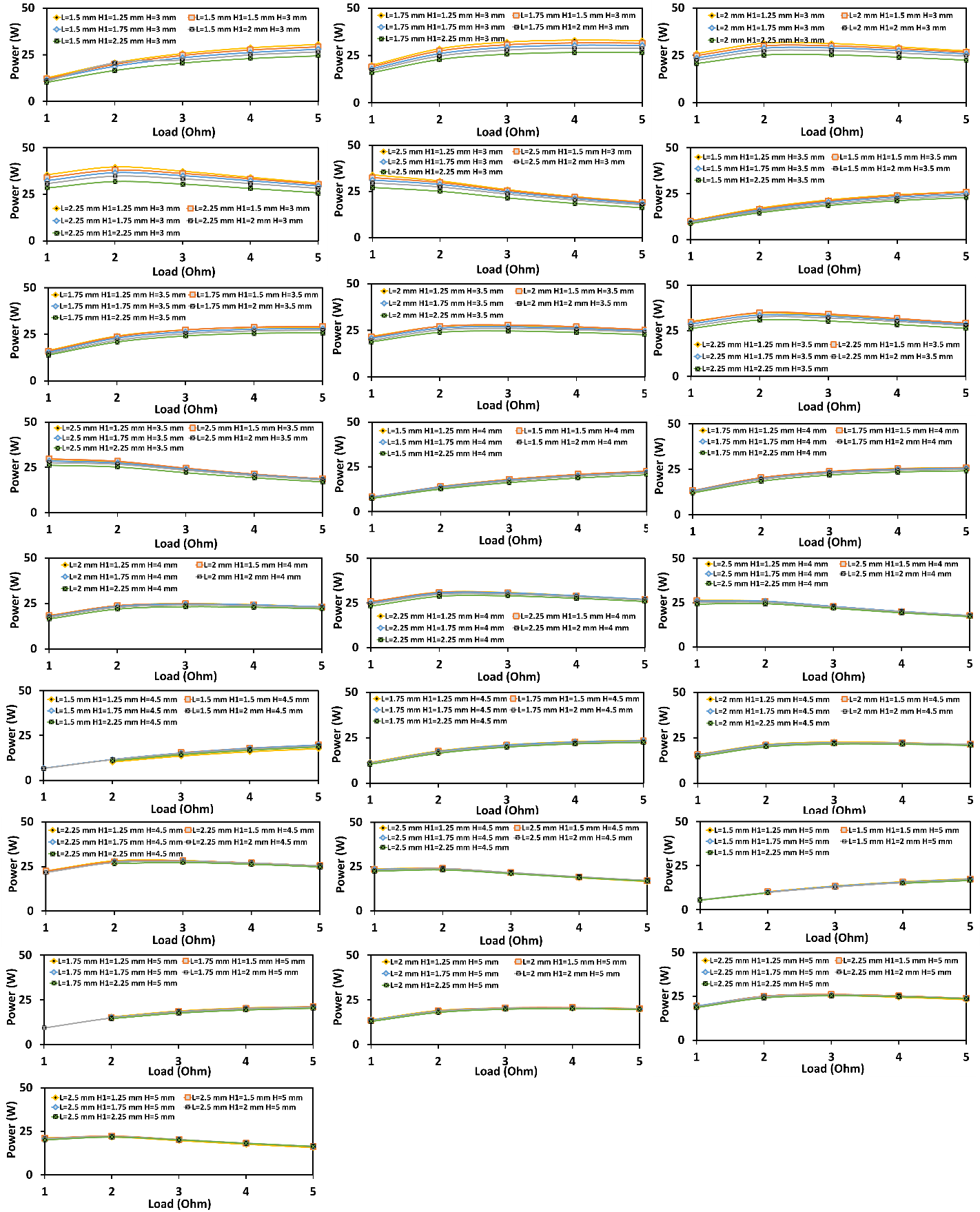


Figure S5. Power output vs. resistive load at different leg dimensions. Hot-side temperature, T_h_ = 873 K and cold-side temperature, T_c_ = 283 K.


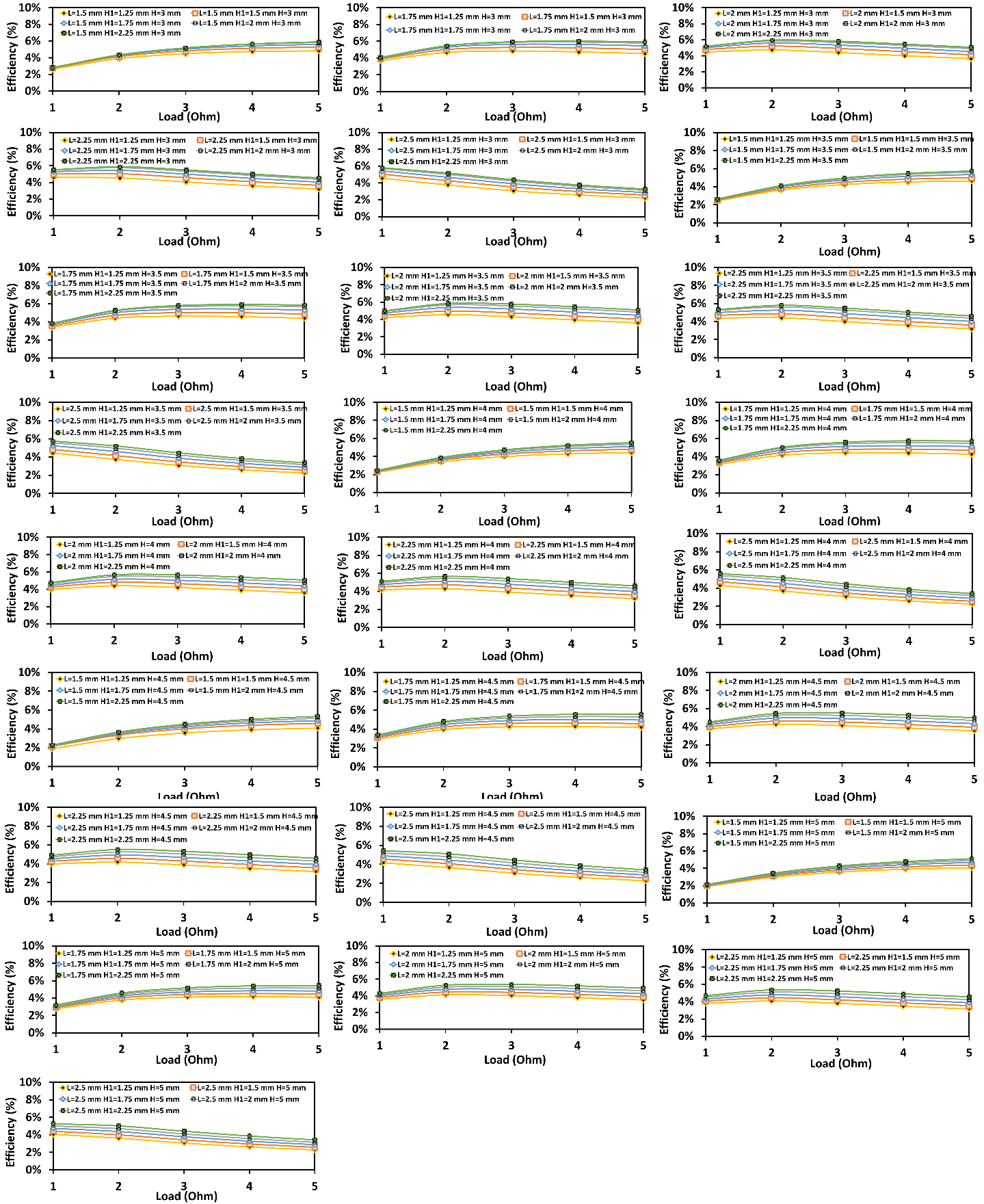


Figure S6. Efficiency vs. resistive load at different legs dimensions. Hot-side temperature, T_h_ = 473 K and cold-side temperature, T_c_ = 283 K.


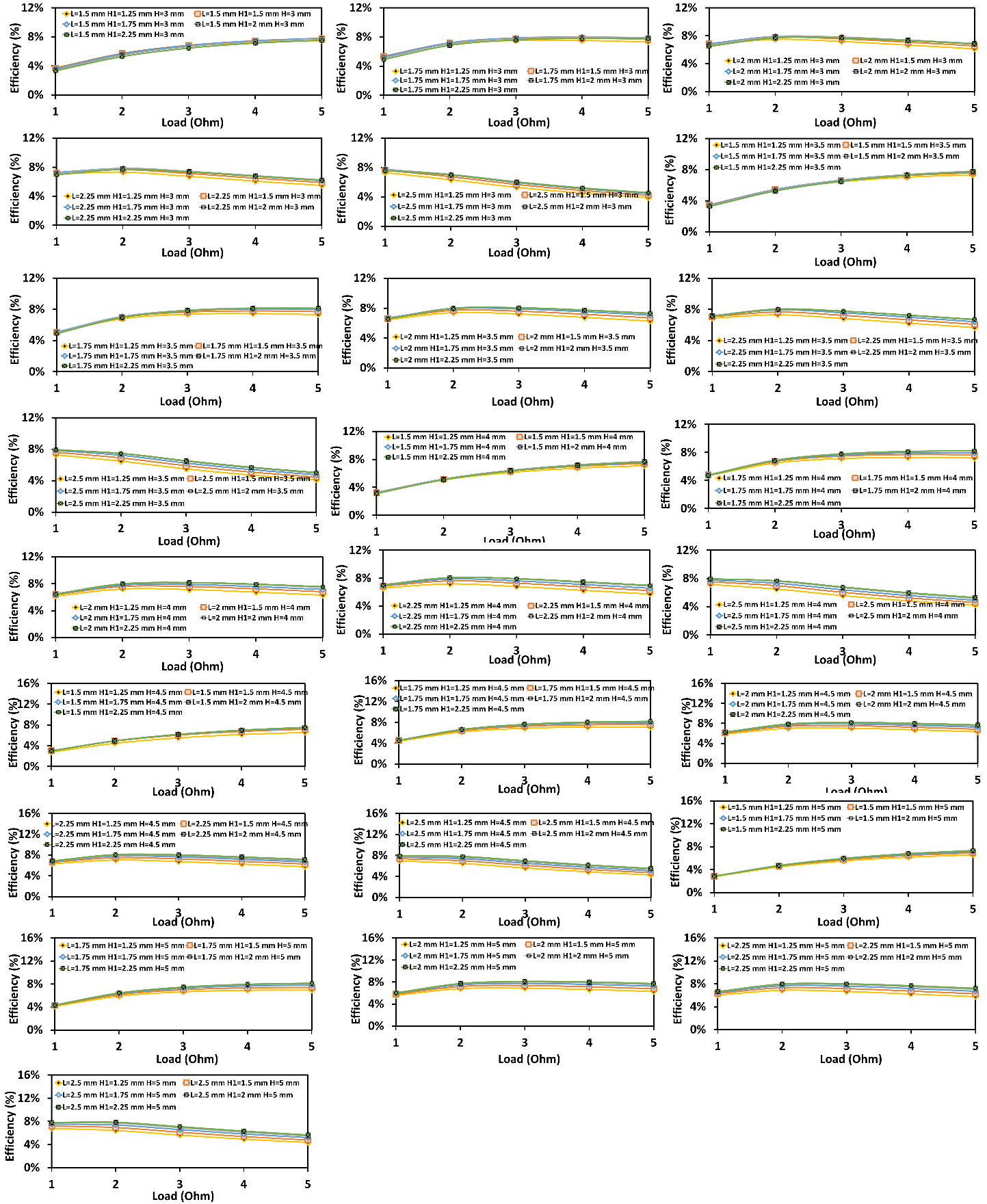


Figure S7. Efficiency vs. resistive load at different leg dimensions. Hot-side temperature, T_h_ = 573 K and cold-side temperature, T_c_ = 283 K.


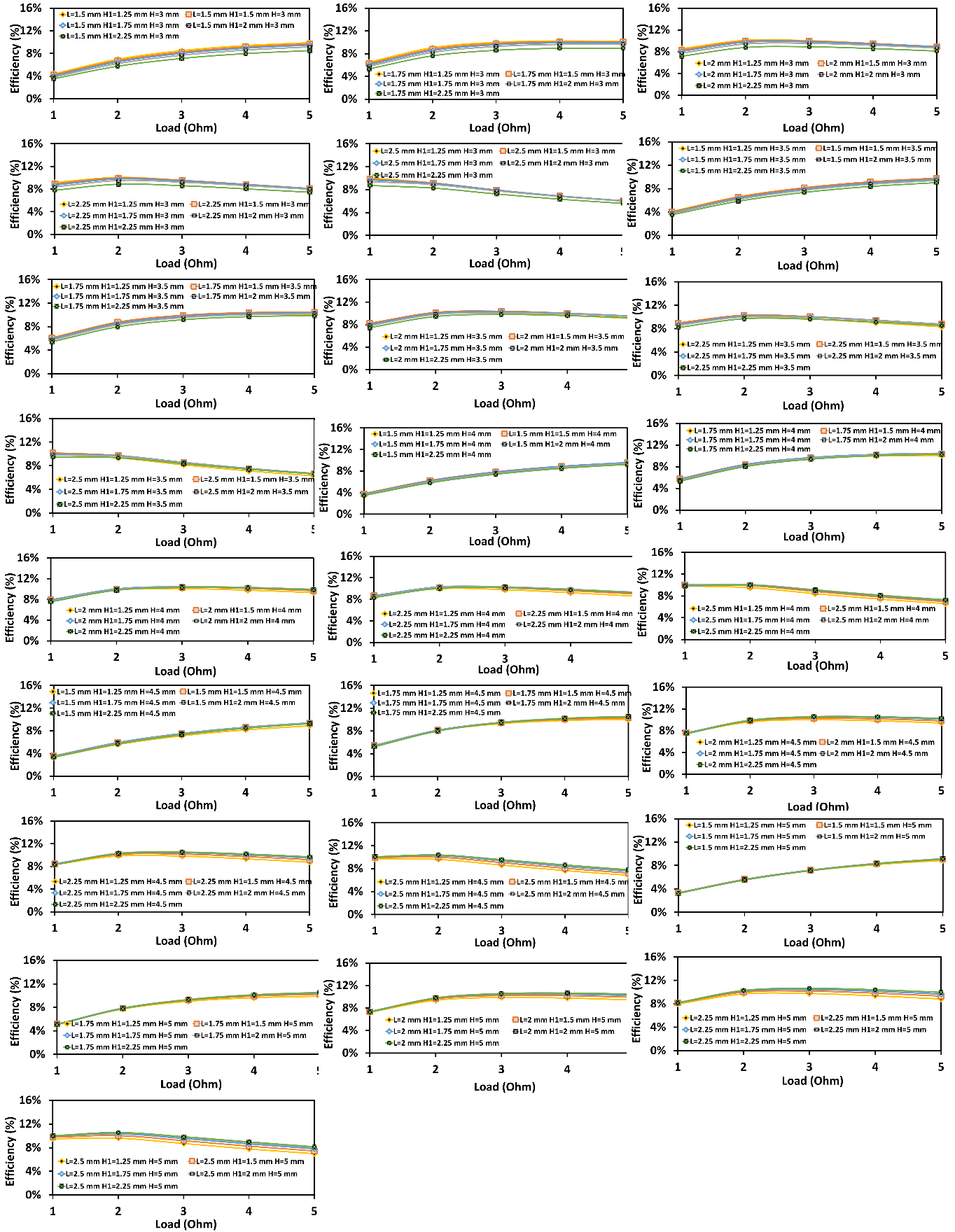


Figure S8. Efficiency vs. resistive load at different leg dimensions. Hot-side temperature, T_h_ = 673 K and cold-side temperature, T_c_ = 283 K.


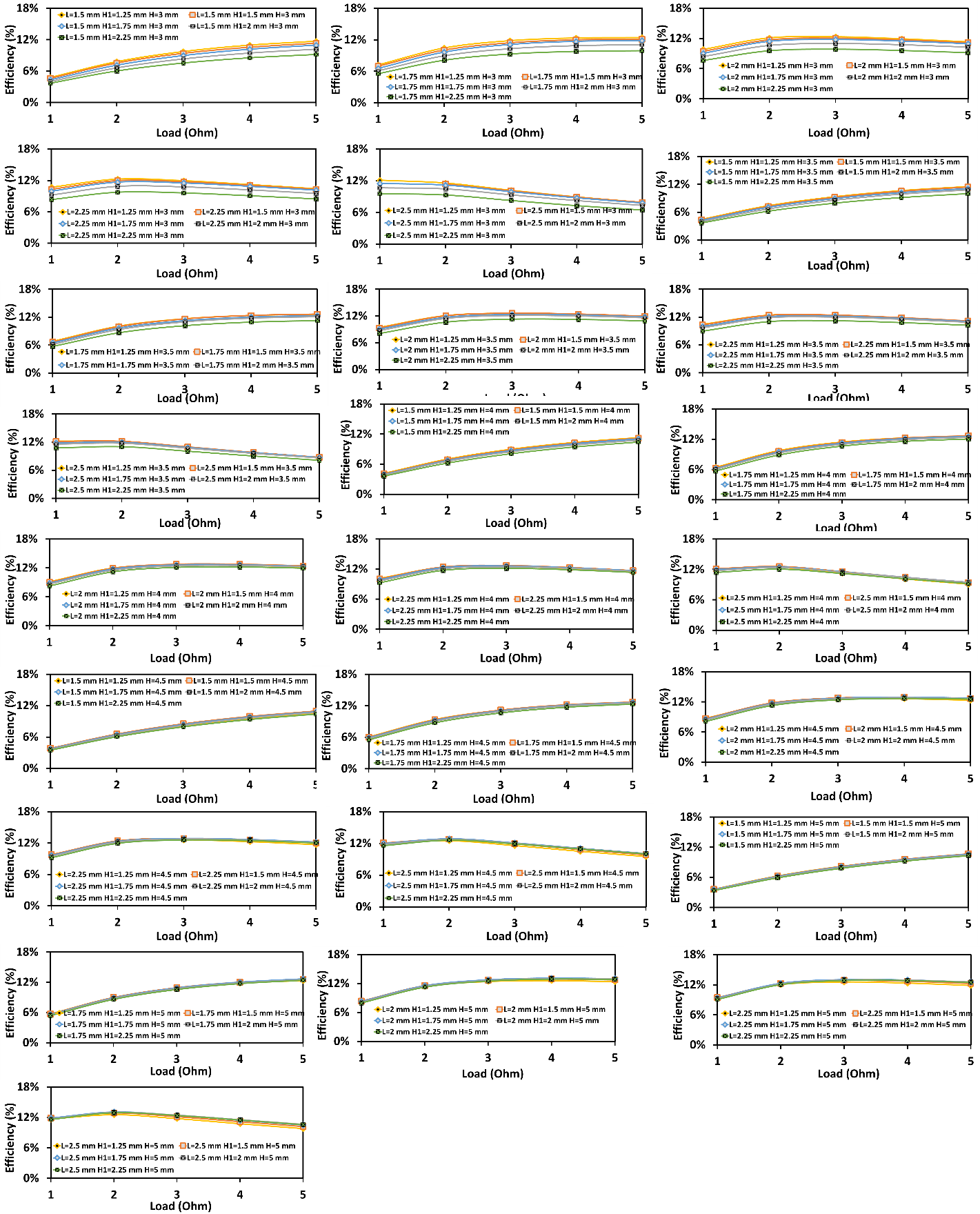


Figure S9. Efficiency vs. resistive load at different leg dimensions. Hot-side temperature, T_h_ = 773 K and cold-side temperature, T_c_ = 283 K.


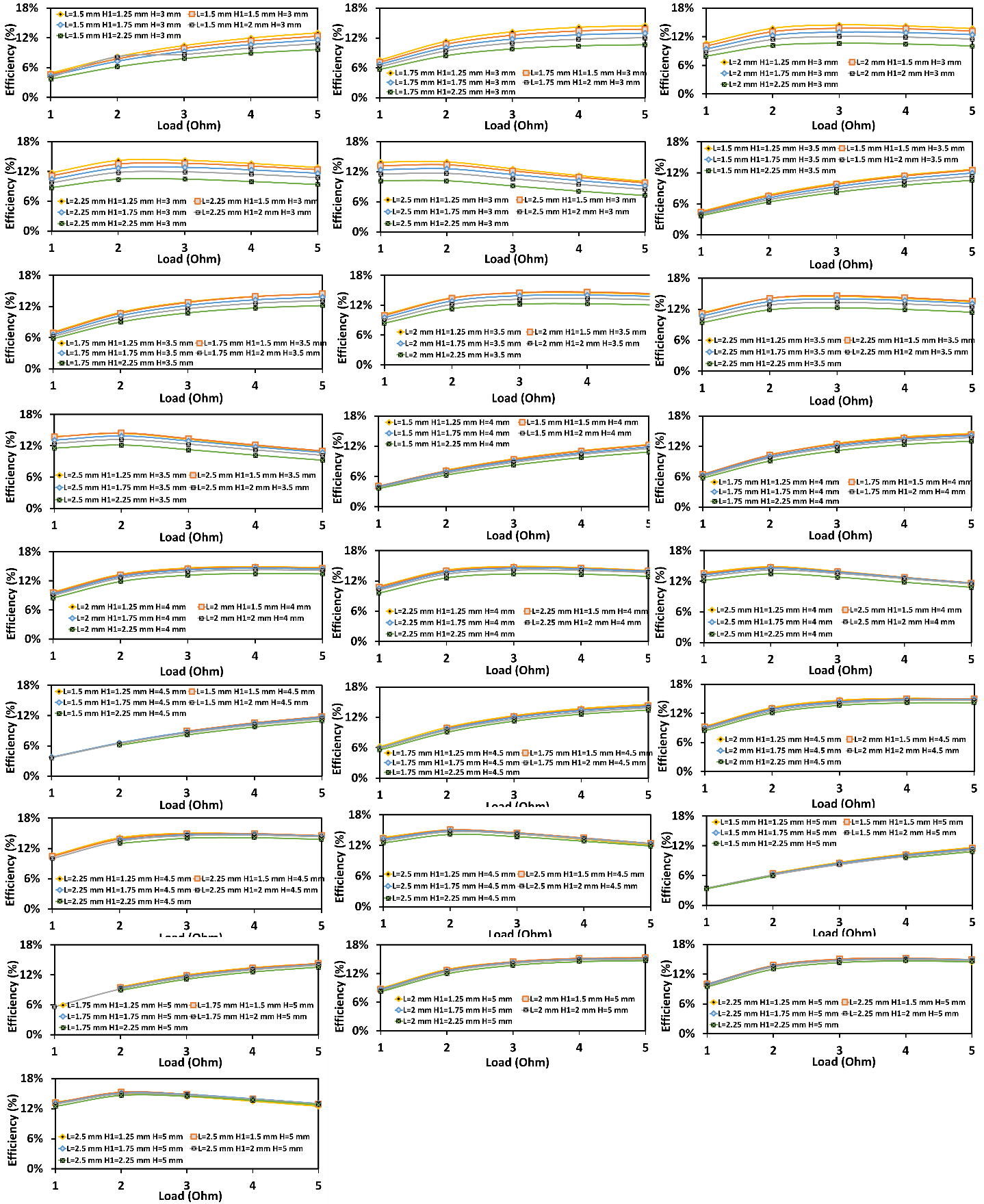


Figure S10. Efficiency vs. resistive load at different leg dimensions. Hot-side temperature, T_h_ = 873 K and cold-side temperature, T_c_ = 283 K.
